# Targeted inhibition of PDGFRA with avapritinib, markedly enhances lenvatinib efficacy in hepatocellular carcinoma in vitro and in vivo: clinical implications

**DOI:** 10.1186/s13046-025-03386-8

**Published:** 2025-05-07

**Authors:** Bixing Zhao, Yang Zhou, Niangmei Cheng, Xiaoyuan Zheng, Geng Chen, Xin Qi, Xiangzhi Zhang, Fei Wang, Qiuyu Zhuang, Yehuda G. Assaraf, Xiaolong Liu, Yingchao Wang, Yongyi Zeng

**Affiliations:** 1 The United Innovation of Mengchao Hepatobiliary Technology Key Laboratory of Fujian Province, Mengchao Hepatobiliary Hospital of Fujian Medical University, Fuzhou, 350025 P. R. China; 2 Mengchao Med-X Center, Fuzhou University, Fuzhou, 350116 P. R. China; 3 The Fred Wyszkowski Cancer Research Laboratory, Faculty of Biology, Technion-Israel Institute of Technology, Haifa, 3200003 Israel

**Keywords:** Hepatocellular carcinoma, Lenvatinib, Resistance, PDGFRA, Avapritinib

## Abstract

**Background:**

Lenvatinib, a tyrosine kinase receptor inhibitor, has emerged as a frontline therapeutic strategy for the management of advanced hepatocellular carcinoma (HCC). However, the modest response rate observed with lenvatinib and the rapid emergence of chemoresistance highlight the urgent need to elucidate the underlying molecular mechanisms. Herein we aimed at identifying the molecular mechanisms underlying lenvatinib resistance in HCC and investigated the efficacy of targeted combination therapies to surmount this chemoresistance.

**Methods:**

We utilized CRISPR/Cas9 gene knockout screening combined with transcriptome sequencing of lenvatinib-resistant HCC cell lines to identify resistance-associated genes. PDGFRA overexpression was validated in human lenvatinib-resistant HCC cells. We further corroborated the in vitro and in vivo role of PDGFRA in lenvatinib resistance using a PDGFRA inhibitor, avapritinib, employing a mouse orthotopic HCC model, patient-derived organoids (PDO), and patient-derived xenografts (PDX). The association between PDGFRA expression and patient prognosis was also assessed. Mechanistic studies were conducted to elucidate the signaling pathways contributing to lenvatinib resistance mediated by PDGFRA.

**Results:**

PDGFRA overexpression was identified as a key determinant of lenvatinib-resistance in HCC cells. Consistently, ectopic PGDGFRA overexpression conferred lenvatinib resistance upon HCC cells. Treatment with the PDGFRA inhibitor avapritinib sensitized HCC cells to lenvatinib in mouse orthotopic HCC, PDO, and PDX models. Increased PDGFRA expression was correlated with poor prognosis in HCC patients. Mechanistic studies revealed that lenvatinib treatment or PDGFRA overexpression promoted HCC resistance through the PTEN/AKT/GSK-3β/β-catenin signaling pathway.

**Conclusions:**

Our findings demonstrate that PDGFRA overexpression mediates lenvatinib resistance in HCC and that targeting PDGFRA with avapritinib, surmounts this resistance. Furthermore, the PTEN/AKT/GSK-3β/β-catenin pathway was implicated in lenvatinib resistance, providing a potential therapeutic strategy for HCC patients displaying lenvatinib resistance. Further clinical studies are warranted to validate these findings and to explore the clinical application of PDGFRA-targeted therapies in HCC treatment.

**Supplementary Information:**

The online version contains supplementary material available at 10.1186/s13046-025-03386-8.

## Background

Lenvatinib, a multikinase inhibitor, targets several receptors, including VEGFR1-VEGFR3, FGFR1-FGFR4, PDGFRα, KIT and RET [1]. Introduction of lenvatinib has been a significant advancement in the treatment of liver cancer by inhibiting angiogenesis, which is crucial for tumor growth and progression [2–4]. Lenvatinib was approved as the first-line treatment of advanced hepatocellular carcinoma (HCC) in the United States, the European Union, Japan, and China [2]. Although a minority of liver cancer patients derive genuine long-term benefits from lenvatinib treatment, the majority do not respond to lenvatinib therapy or gradually acquire resistance [3]. Therefore, deciphering the molecular mechanisms underlying anticancer drug resistance and developing efficacious combination therapies to enhance the sensitivity of liver cancer patients to lenvatinib, is of paramount importance to further improve patient outcomes [2, 3]. A series of recent studies have reported the mechanisms underlying the acquisition of lenvatinib resistance in tumor therapy, which are related to the regulation of cell death or proliferation, histological transformation, metabolism, transport processes, and epigenetics [5–7].

Platelet Derived Growth Factor Receptor A (PDGFRA) is a central receptor present on the surface of a variety of cell types [8]. This receptor is encoded by the PDGFRA gene in humans. Upon binding of specific isoforms of platelet-derived growth factors (PDGFs) to PDGFRA, the receptor becomes activated, thereby initiating cell signaling pathways that promote cellular growth, differentiation, and other responses [9]. PDGFRA plays a pivotal role in the embryonic development of various tissues and organs, and it is essential for the ongoing maintenance of these tissue and structures, especially those related to hematopoietic tissues [10]. Mutations within the PDGFRA gene are associated with a spectrum of neoplasms, most notably including the clonal hypereosinophilia class of malignancies and gastrointestinal stromal tumors (GISTs) [11].

Avapritinib is the only potent and selective inhibitor approved for the treatment of GISTs harboring the most common primary mutation D842V of PDGFRα, encoded by the PDGFRA gene [12]. The FDA approval of avapritinib was based upon the NAVIGATOR clinical trial, which revealed overall response rates of 88–91% [13, 14]. However, in other types of tumors including liver cancer, the efficacy of avapritinib remains unknown.

In this study, we found that PDGFRA is significantly upregulated in HCC cells displaying Lenvatinib- resistance. Loss-and-gain of function studies have shown that PDGFRA is a key mediator of HCC resistance to lenvatinib. More importantly, targeting PDGFRA with its selective inhibitor avapritinib, significantly enhanced the sensitivity of liver cancer cells to lenvatinib, a result that has also been verified in patient-derived organoid (PDO) models and patient-derived xenograft (PDX) mouse models. Furthermore, clinical data also indicated that patients with low PDGFRA expression exhibit a better therapeutic response to lenvatinib. Thus, avapritinib emerges as a therapeutic agent capable of restoring lenvatinib sensitivity in drug resistant HCC.

## Materials and methods

### Human specimens

HCC tissues were obtained from patients who underwent curative surgery at Mengchao Hepatobiliary Hospital of Fujian Medical University. Sample collection and use were approved by Medical Ethics Committee of Mengchao Hepatobiliary Hospital of Fujian Medical University (2022_018_01). Meanwhile, informed consent was provided by the patients.

### Cell line

The human HCC cell line SNU-449, Huh7, SK-Hep-1, Hep3B, SNU-398 and C3A were obtained from the American Type Culture Collection (ATCC, USA). The mouse HCC cell line Hepa1-6 and human HCC SMMC-7721 cells were acquired from the cell bank of the National Collection of Authenticated Cell Cultures, China. All cells were cultured in a complete medium (MEM, DMEM, RPMI-1640) (Hyclone, USA) supplemented with 10% fetal bovine serum (Hyclone, USA) at 37ºC, 5% CO_2_.

### CRISPR library screening for genes mediating lenvatinib resistance

The GeCKO v2 CRISPR library (Addgene, USA) was employed to identify genes implicated in lenvatinib resistance. In accordance with established protocols, the library plasmids were subjected to transformation and extraction, complemented by rigorous quality control measures. RNA sequencing was conducted to ascertain the integrity of the library plasmids. Additionally, the lentiviral plasmid lenti-Cas9-blast was procured from Addgene (USA) and utilized for the generation of lentiviruses expressing Cas9. SNU-449 or SMMC-7721 cells were infected with these lentiviruses at a multiplicity of infection (MOI) of 10. Following infection, blasticidin selection was applied after 48 h, and Western blot analysis was performed to verify the overexpression of the Cas9 protein. Upon reaching a cell density of approximately 10%, puromycin was introduced at varying concentrations (0, 1, 2, 3, 4, 5, 6, 7, and 8 µg/ml), with the culture medium being refreshed every three days. Following a six-day incubation, cell viability was assessed using the CCK8 assay to ascertain the minimum antibiotic concentration necessary to eliminate all wild-type cells. The sgRNA library lentivirus was packaged while adhering to the same protocol as the Cas9 lentivirus preparation, and the lentiviral titer was determined. SNU-449-Cas9 cells were then stably transduced with the sgRNA library lentivirus at an MOI of 0.3 in the presence of polybrene, followed by puromycin selection for seven days. The surviving SNU-449 cells were designated as CRISPR library cells and were subjected to sequential screening with increasing concentrations of lenvatinib (10, 15, 20 and 30 µM) to identify genes associated with lenvatinib resistance.

### Establishment of lenvatinib-resistant cell lines

An intermittent drug induction method was employed to establish lenvatinib-resistant cell lines. Initially, the half-maximal inhibitory concentration (IC_50_) of lenvatinib for the wild-type liver cancer cell lines SNU-449 and SMMC-7721 was determined using the Cell Counting Kit-8 (CCK-8) assay. Thereafter, cells in the logarithmic growth phase were progressively exposed to increasing concentrations of lenvatinib. After 12 weeks of continuous cultivation, the IC_50_ values for the newly established resistant cell lines were determined. The two lenvatinib-resistant cell lines were then maintained through continuous culture in the presence of lenvatinib, ensuring their sustained resistance phenotype.

### Transcriptome sequencing

Transcriptome sequencing of the cells was conducted at Berry Hekang Biotechnology Corporation (Beijing, China). Total cellular RNA was extracted, followed by a stringent quality assessment to evaluate the purity, concentration, and integrity of the RNA. mRNA was isolated through hybridization to oligo dT magnetic beads and then subjected to controlled fragmentation. The fragmented mRNA served as a template for reverse transcription using either random hexamers or oligo dT primers, yielding cDNA. The cDNA library was subsequently sequenced employing a high-throughput sequencing platform, yielding an abundance of sequence data. Then, a thorough differential expression analysis was performed to delineate the transcriptomic alterations in lenvatinib-resistant cell lines.

### Overexpression and knockdown cell lines

PCDH-CMV-3xFlag-copGFP-puro was chosen for lentivirus packaging for PDGFRA overexpression. sgRNAs targeting PDGFRA were designed to transfect pBOB-CAS9 and screened by puromycin. The sgPDGFRA primers were as follows:

sgPDGFRA-1: F: CACCGAAAGCCCTGTCTGCTGTCGT,

R: AAACACGACAGCAGACAGGGCTTTC,

sgPDGFRA-2: F: CACCGTCGGGATCAGTTGTGCGACA,

R: AAACTGTCGCACAACTGATCCCGAC.

All selected cell lines were assessed for the expression levels of the target gene PDGFRA using the Western blotting assay.

### Quantitative PCR (qPCR)

Total RNAs were extracted using TransZol Up Plus RNA kit (Beijing TransGenBiotech, China) and quantified by Nanodrop 2000 (ThermoFisher, USA). 1 µg of total RNA was reverse-transcribed into cDNA by Transcriptor First Strand cDNA Synthesis Kit (Roche Ltd., Basel, Switzerland). Quantitative PCR was performed with SYBR Green qPCR Master mix (DBI-2233, DBI, Germany). Human 18 S rRNA was used as endogenous control. RT-qPCR was carried out under the following conditions: 95 °C for 5 min, 40 cycles at 95 °C for 10 s, 60 °C for 30 s and 72 °C for 30 s. The relative expression of RNA was calculated using the 2-ΔΔCt method.

The qPCR primer sequences were as follow:

PDGFRA-F: (5’-3’) GACTTTCGCCAAAGTGGAGGAG;

PDGFRA-R: (5’-3’) AGCCACCGTGAGTTCAGAACGC.

### Western blot analysis

Total proteins were extracted by using RIPA buffer (Beyotime) supplemented with 1% protease inhibitor cocktails (Roche), and the concentration of proteins was measured using the BCA Protein Assay Kit (Transgene). The proteins were loaded and resolved on 10% SDS-PAGE (Bio-Rad) and transferred to nitrocellulose membranes (PALL Corporation). After blocking in 5% skim milk for 1 h at room temperature, the membranes were incubated with primary antibodies overnight at 4ºC. The membranes were incubated with horseradish peroxidase (HRP)-conjugated secondary antibodies (Abcam) for 1 h at room temperature. The blot signals were visualized using ECL reagent (ThermoFisher) and detected using the ChemiDoc MP Imaging System (Bio-Rad).The primary antibodies were as follows: β-actin (ab115777; Abcam), mTOR (2983P, CST), p-mTOR (5536P, CST), AKT (4691p, CST), p-AKT (4060p, CST), MEK (9122, CST), p-MEK (9121, CST), ERK (ab17942, abcam), p-ERK (4370, CST), PTEN (9188,CST), GSK3β (12456, CST), p-GSK3β (9323,CST), GADPH (5174T, CST), PDGFRA (60045-1-lg, proteintech), and β-catenin (8480p, CST),

### Colony formation assay

The cell lines were seeded in a six-well plate (2 × 10^3^ cells/well), ensuring a uniform distribution of cells across the surface. Then, the plates were cultured with complete medium at 37 °C and 5% (vol/vol) CO_2_. The cells were then allowed to adhere and grown for a period of 10–14 days. After the incubation period, cells were stained with 0.5% crystal violet to visualize the clones. Photographs were captured, and the number of colonies was counted using Image J software.

### Patient-derived organoids (PDO)

Firstly, the tissues derived from patients were mechanically cut into small pieces of 1-3mm^3^. Then the tissue fragments were digested with 10mL of Tumor Tissue Digestion Solution (K601003, bioGenous^TM^) in a 15mL conical tube at 37 °C, with variable incubation times ranging from 15 to 45 min. The digestion was terminated with FBS (10%) addition. Then, the suspension was filtered with 100 μm strainer and centrifuged for 3 min at 300 g. Aspirate The supernatant was aspirated and the pellet was resuspended in ECM (M315066, bioGenous^TM^). The PDOs were seeded in 24-well plates. The plates were placed into a humidified incubator at 37 °C and 5% CO_2_ for 15–25 min to let the ECM solidify. Organoid complete medium (K2105-HCC, bioGenous^TM^) was added to each well. Organoids were passaged after dissociation with Organoid Dissociation Solution (E238001, bioGenous^TM^). For storage, the organoids were resuspended in Organoid Cryopreservation Medium (E238023, bioGenous^TM^) and frozen following standard procedures.

To evaluate the cell killing effect of avapritinib and lenvatinib in PDOs, the PDOs were seeded in 96-well plates and further incubated for 24 h. Thereafter, complete growth medium containing increasing concentrations of avapritinib and lenvatinib were added into the PDOs. Then, the PDOs were incubated for another 72 h. Afterwards, the PDOs were stained with Calcein-AM and propidium iodide (PI) for 30 min and then imaged by confocal laser scanning microscope. The cytotoxicity of avapritinib and lenvatinib in PDO was quantified by CellTiter-Glo^®^3D Cell Viability Assay (Promega, G9681). CellTiter-Glo^®^3D Reagent was added to the PDOs, and shaking for 5 min was performed. After 25 min incubation, the chemiluminescence was determined using a microplate reader according to the manufacturer instructions.

### Animal experimentation

All animal experiments were conducted in strict accordance with protocols approved by the Animal Ethics Committee of Mengchao Hepatobiliary Hospital of Fujian Medical University (MCHH-AEC-2022-12). For the subcutaneous tumor model, wild-type SMMC-7721 cells and lenvatinib-resistant SMMC-7721 cells (5 × 10^6^cells/mouse) were subcutaneously injected into the right axilla of 6-week-old male NCG mice (*n* = 5 per group). Tumor volume was determined using caliper measurements and calculated using the modified ellipsoidal formula: tumor volume = 0.5×length×width^2^. Upon reaching a tumor volume of approximately 200 mm^3^, mice were randomly assigned to one of three treatment groups, with treatments administered five days per week: those implanted with wild-type SMMC-7721 cells received either vehicle control or lenvatinib (4 mg/kg, orally), whereas those with lenvatinib-resistant cells were treated with lenvatinib (4 mg/kg, orally).

For further assessment, SMMC-7721 lenvatinib-resistant cells (5 × 10^6^ cells/mouse) were subcutaneously injected into the right axilla of 6-week-old male NCG mice (*n* = 5 per group). Once the tumor volume reached approximately 200 mm^3, mice were randomly assigned to receive treatment five days per week with either vehicle, lenvatinib (4 mg/kg, oral gavage), avapritinib (10 mg/kg, oral gavage), or a combination of both drugs, administered at the same dosage and schedule as monotherapy. In addition, SMMC-7721 cells overexpressing PDGFRA were implanted subcutaneously in NCG mice, following the same methodology. After tumor establishment, these mice were similarly assigned to the corresponding treatment groups.

For orthotopic tumor model, male C57L/J mice (8–10 weeks old, *n* = 10 per group) were orthotopically implanted with 5 × 10^5^ Hepa1-6 cells. The cells were suspended in a 25 µl mixture of serum-free DMEM and Matrigel (BD Biosciences) at a 1:1 ratio. Under isoflurane anesthesia, an 8-mm transverse incision was made in the upper abdomen, and the cell suspension was injected directly into the left hepatic lobe using a microsyringe. Mice implanted with Hepa1-6 cells or PDGFRA-overexpressing Hepa1-6 cells were randomly assigned to receive either vehicle control or lenvatinib (4 mg/kg, via oral gavage) for three weeks. For mice implanted with PDGFRA-overexpressing Hepa1-6 cells, treatment was administered five days per week with either PBS, lenvatinib (4 mg/kg), avapritinib (10 mg/kg), or a combination of both drugs, following the same dosing and scheduling as the monotherapy. After three weeks of treatment, half of the mice were euthanized.

Body weight was monitored throughout the treatment period, and tumor weight was measured at the study’s endpoint. Tumor fluorescence intensity was monitored using the IVIS Spectrum Animal Imaging System (PerkinElmer, USA). For survival analysis, treatment continued until the tumor fluorescence intensity reached 10^9^.

### Patient-derived xenografts (PDXs)

Surgically resected tumor tissues from HCC patients were utilized for xenotransplantation following informed consent and approval by the Medical Ethics Committee of Mengchao Hepatobiliary Hospital of Fujian Medical University (MCHH-AEC-2022-12). Patient-derived samples were collected, trimmed, and sectioned into fragments measuring 20–30 mm³. These fragments were subcutaneously implanted into the right axilla of anesthetized, 6–8-week-old male NCG mice within two hours post-resection. Tumor growth was monitored every three days using calipers, and the establishment of PDXs in each mouse was confirmed over a minimum period of three months. Once the tumors reached a volume of 1000 mm³, the mice were euthanized, and tumor fragments were excised for implantation into the right axillary region of subsequent generations of NCG mice. Tumors were passaged once before being implanted subcutaneously into new NCG mice. When the tumor volume approximated 200 mm³, the mice were randomly assigned to receive treatment five days per week with either vehicle control, lenvatinib (4 mg/kg, via oral gavage), avapritinib (10 mg/kg, via oral gavage), or a combination of both drugs, following the same dosage and schedule as the monotherapy.

### Hematoxylin and eosin staining and immunohistochemistry

Tumor samples were formalin-fixed, paraffin-embedded, and sectioned at 4 μm thickness for staining with hematoxylin and eosin (H&E) and immunohistochemistry.

Immunohistochemical analysis was performed using the PDGFRA antibody (sc-398206, Santa Cruz) on formalin-fixed paraffin-embedded HCC samples. Xenografted tumors were similarly probed with antibodies against Ki-67 (D2H10, CST) and PDGFRA. After primary antibody incubation, positive staining was visualized using DAB + as a chromogen.

Quantitative analysis was conducted using QuPath (0.4.3) software, assessing the percentage of positively stained cells and staining intensity per high-power field in representative sections. H-score assessment was also performed using QuPath (0.4.3). Membrane PDGFRA staining was categorized and scored as follows: 0 for no staining, 1 + for light staining visible at high magnification, 2 + for intermediate staining, and 3 + for dark staining visible at low magnification. The H-score was calculated using the formula: 1×(% of 1 + cells) + 2×(% of 2 + cells) + 3×(% of 3 + cells). Each patient was assigned a score from 0 to 300, with a threshold of 200 used for discrimination. An H-score below 109.5 indicated low PDGFRA expression, while an H-score of 109.5 or above was considered high PDGFRA expression.

### Statistical analysis

All data analyses were performed using GraphPad (version 8.0). The data are presented as the mean ± SD with minimally three independent replicates. Statistical analyses of normally distributed variables were performed using the Student’s t test, and analyses of data with skewed distributions were performed using the Mann-Whitney U test.

## Results

### CRISPR/Cas9 library screening of genes associated with lenvatinib resistance

**Fig. 1:**
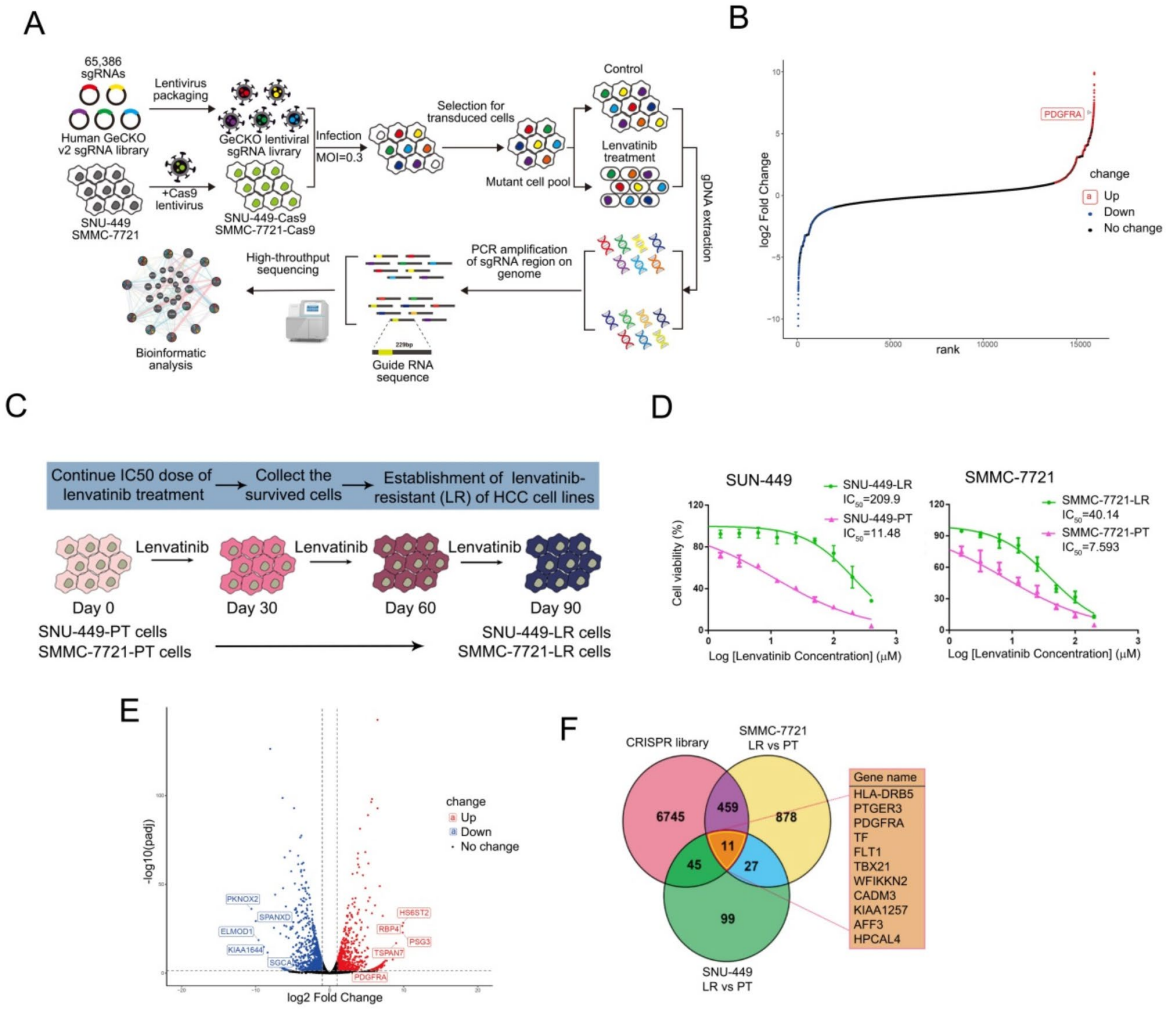
CRISPR/Cas9 knockout library screening and transcriptome sequencing identifies PDGFRA as a potential driver of lenvatinib resistance. (**A**) Workflow of genome-wide CRISPR/Cas9 knockout library screening. (**B**) The distribution of normalized CRISPR viability scores (lenvatinib treatment versus control) for genes present in the sgRNA library. (**C**) A flow chart of the Establishment of lenvatinib resistant HCC sublines. (**D**) Half maximal inhibitory lenvatinib concentration curves of parental (PT) HCC cells and lenvatinib resistant (LR) cells. (**E**) Volcano plot depicting the differentially expressed protein profiles between PT and LR cells. (**F**) A Venn diagram illustrating the intersection of genes identified from CRISPR/Cas9 library screening and transcriptomic sequencing results

To identify genes associated with resistance to lenvatinib, we conducted a genome-wide knockout screen using a CRISPR/Cas9 library (GeCKO v2), which contains 65,386 sgRNAs targeting 19,052 protein-coding genes and 1,864 microRNAs [15]. We first constructed SNU-449 and SMMC-7721 cell lines that overexpress Cas9. The library was infected into these tumor cells at a low multiplicity of infection (MOI), followed by a 7-day selection with lenvatinib at the 50% inhibitory concentration (IC_50_) (11 µM in SMMC-7721 and 7 µM in SNU-449 cells) (Supplementary Figure S1). Genomic DNA from the surviving cells was extracted and subjected to PCR and the PCR products were analyzed by high-throughput sequencing and enrichment analysis was performed. Thereafter, bioinformatics analysis was undertaken to screen for genes that may be associated with lenvatinib resistance (Fig. 1A). From the CRISPR/Cas9 library screening, we identified a subset of sgRNAs targeting 11,412 genes which were significantly depleted in lenvatinib treated cells when compared to control cells, indicating that these genes might be potential drivers of lenvatinib resistance (Fig. 1B).

In parallel to the CRISPR/Cas9 knockout library screening, we also conducted RNA sequencing to pin-point the transcriptomic changes in lenvatinib-resistant cells. We established sublines with acquired resistance to clinically relevant lenvantinib concentrations representative of the drug plasma levels (∼ 100 nM) [16] observed in HCC patients. Hence, two human HCC cell lines, SNU-449 and SMMC-7721, were exposed to a constant lenvatinib concentration representing the IC_50_ value for a total duration of three months, resulting in the lenvatinib resistant cell lines SNU-449-LR and SMMC-7721-LR. The lenvantinib drug selection protocol is depicted in Fig. 1C. The lenvatinib-resistant sublines were then subjected to a growth inhibition assay in increasing concentrations of lenvatinib to determine their lenvatinib-resistance levels (Fig. 1D). Subsequently, parental (PT) SNU-449 and SMMC-7721 cells, as well as the lenvatinib-resistant (LR) cells, were subjected to RNA sequencing to identify differentially expressed genes. A total of 1519 genes were found to be upregulated in lenvatinib-resistant cells, among which 38 genes were upregulated simultaneously in the two drug resistant cell lines (Fig. 1E-F). The transcriptomic data from the two lenvatinib-resistant cell lines were intersected with the results obtained from the CRISPR/Cas9 knockout library screening, ultimately yielding 11 genes potentially associated with lenvatinib-resistance, among which the PDGFRA gene attracted our attention, as being a *bona fide* target of lenvantinib (Fig. 1F).

### Lenvatinib resistance in HCC cells is acquired via PDGFRA overexpression

**Fig. 2:**
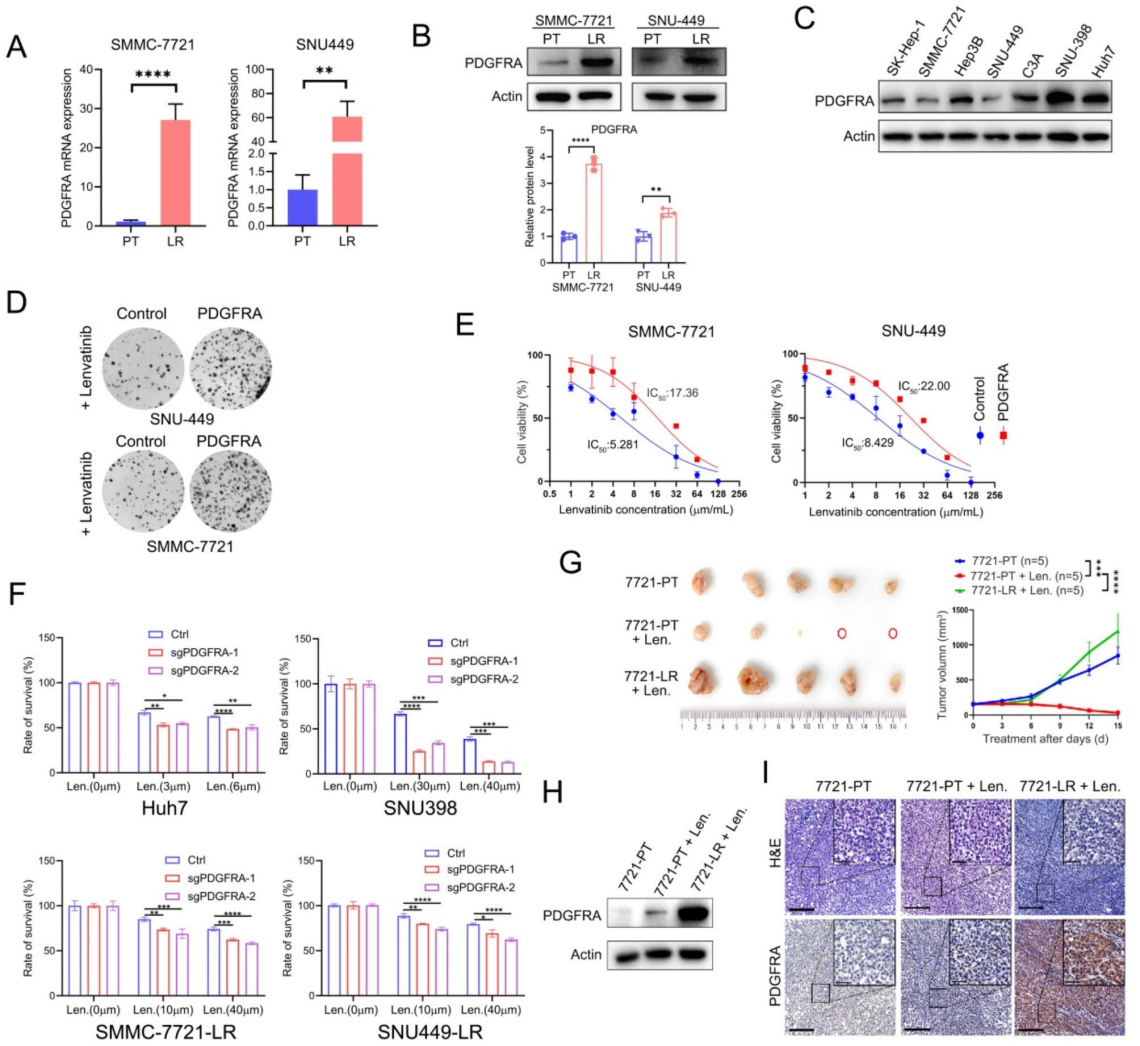
Lenvatinib resistance in HCC cells is acquired via PDGFRA overexpression. (**A**) qPCR was used to detect the mRNA levels of PDGFR in the SMMC-7721-PT, SMMC-7721-LR, SNU-449-PT and SNU-449-LR cells. (**B**) Western blot analysis was conducted to assess the protein expression levels of PDGFRA in the four cell lines. (**C**) Western blot analysis of PDGFRA protein levels in the different HCC cell lines. (**D**) The colony formation capacity of PDGFRA-overexpressing and control SNU-449 and SMMC-7721 cells was assessed by a clonogenic assay following lenvatinib treatment. (**E**) SMMC-7721/SNU-449 cells overexpressing PDGFRA and control cells were treated with lenvatinib, and their cell survival curves were depicted. (**F**) PDGFRA was knocked down in Huh7, SNU-398, SMMC-7721-LR, and SNU-449-LR cells, and cell survival was assessed after treatment with various concentrations of lenvatinib. (**G**) Representative xenograft tumors and tumor growth curves for the following three groups at the endpoint in a subcutaneous implantation mouse model: SMMC-7721-PT group, SMMC-7721-PT plus lenvatinib group, and SMMC-7721-LR plus lenvatinib group (*N* = 5 for each group). (**H**) Western blot analysis was used to determine the levels of PDGFRA protein in the subcutaneous tumors from different groups. (**I**) Representative H&E staining and IHC images of PDGFRA in the subcutaneous implantation mouse model

To validate the sequencing data, we employed qPCR and Western blot analysis to assess the differential mRNA and protein levels of PDGFRA in PT and LR cells. PDGFRA expression was significantly upregulated in LR SMMC-7721 and SNU-449 cells both at the mRNA and protein levels (Fig. 2A, B). To elucidate the mechanisms underlying the upregulation of PDGFRA in LR cells, we conducted additional analyses to assess alterations in PDGFRA gene copy number and the possible presence of PDGFRA mutations in these resistant cells. To our surprise, in LR cells, neither mutations nor significant copy number variations were observed in PDGFRA (Supplementary Figure S2). Additionally, we examined the expression levels of the PDGFRA homologue, PDGFRB, in LR cells. Our findings demonstrated that there was no significant alteration in the expression levels of the PDGFRB protein in LR cells (Supplementary Figure S3).

To further clarify whether PDGFRA is involved in the resistance of HCC cells to lenvatinib, we initially examined PDGFRA expression across various HCC cell lines; clearly, some HCC cell lines displayed intrinsically elevated PDGFRA levels when compared to other cell lines (Fig. 2C). Remarkably, ectopic overexpression of PDGFRA in PDGFRA-low-expressing SMMC-7721 and SNU-449 cell lines was found to diminish the sensitivity of these cells to lenvatinib (Figs. 2D, E). Conversely, knockdown of PDGFRA in Huh7 and SNU-398 cells highly overexpression PDGFRA significantly enhanced their sensitivity to the drug (Figs. 2F). Additionally, in LR stant cell lines, knockdown of PDGFRA restored the sensitivity to lenvatinib (Fig. 2F). These findings indicate that PDGFRA is a central contributing factor to lenvatinib resistance in liver cancer cells.

Building on these in vitro results, we further validated our findings using an in vivo mouse xenograft tumor model. The results demonstrated that subcutaneous HCC tumor models established with LR cells, exhibited markedly reduced sensitivity to lenvatinib compared to PT cells in the animal model (Fig. 2G). Consistent with the in vitro findings, a significant upregulation of PDGFRA was also detected in the tumor specimens (Fig. 2H, I). Collectively, these findings suggest that PDGFRA plays an important role in lenvatinib-resistance in HCC cells.

### Avapritinib, a PDGFRA inhibitor sensitizes HCC cells to lenvatinib treatment

**Fig. 3:**
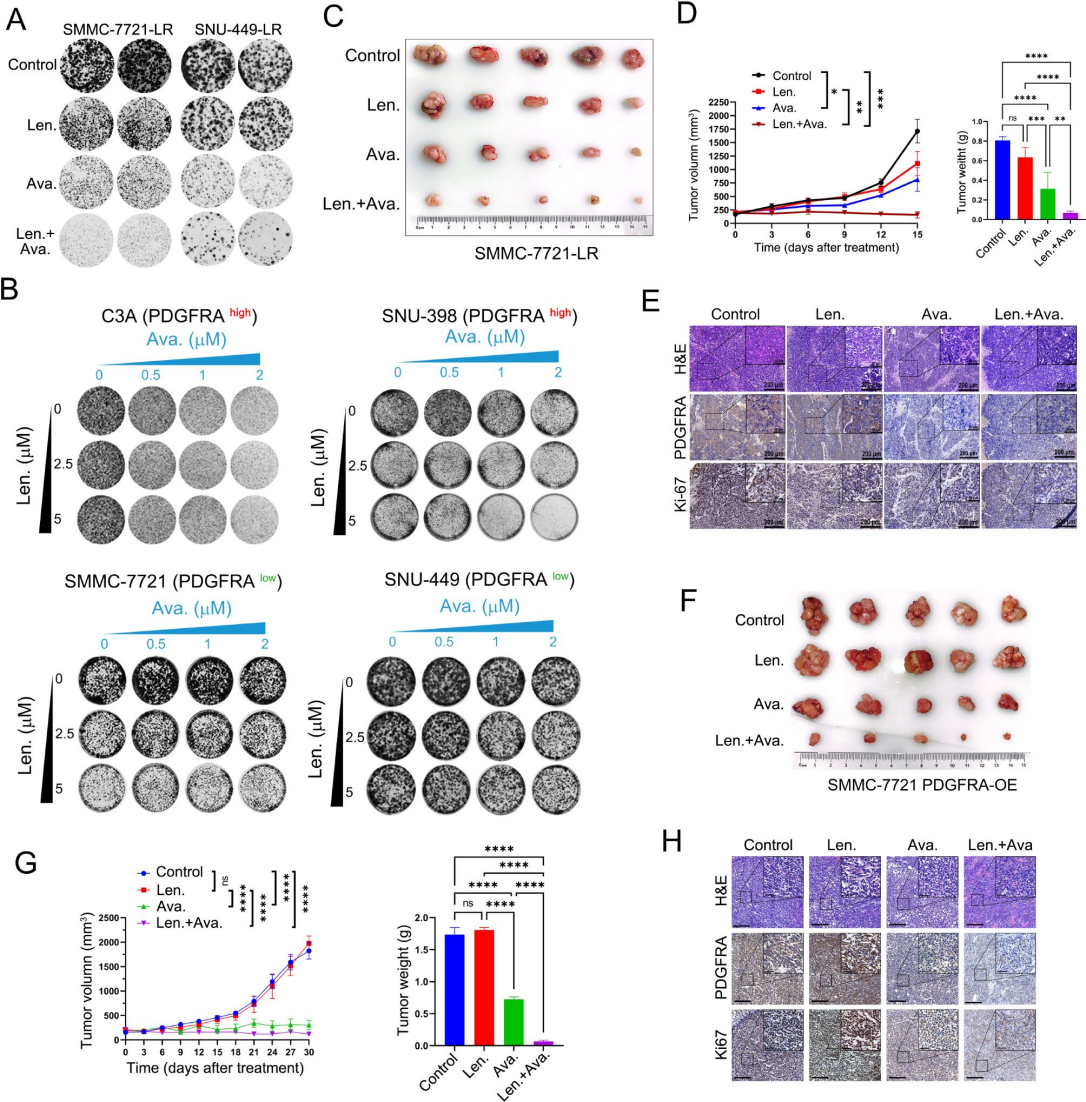
PDGFRA inhibitor avapritinib sensitizes HCC cells to lenvatinib treatment. (**A**) The effect of lenvatinib and avapritinib was tested in the colony-formation assay in SMMC-7721-LR and SNU-449-LR cells. (**B**) Synergistic response to the combination of lenvatinib and avapritinib in PDGFRA high C3A and SNU-398 cells and in PDGFRA low SMMC-7721 and SNU-449 cells. (**C**) Representative tumor images of each group of SMMC-7721-LR xenografts at the end of lenvatinib, avapritinib or combination treatment. (**D**) Tumor growth curves and tumor weight of each group are shown. (**E**) Representative H&E staining, IHC images of PDGFRA and Ki67 in subcutaneous implantation mouse model. (**F**) Representative tumor images of each group of PDGFRA overexpressing SMMC-7721 xenografts at the end of lenvatinib, avapritinib or combination treatment. (**G**) Tumor growth curve and tumor weight of each group (**H**) Representative H&E staining, IHC images of PDGFRA and Ki67 in subcutaneous implantation mouse model

Given that PDGFRA mediates resistance to lenvatinib in HCC cells, a question arose whether targeted inhibition of PDGFRA could reverse lenvatinib resistance in HCC. Consequently, we hypothesized that the combination of the PDGFRA-targeted inhibitor avapritinib with lenvatinib might exhibit synergistic inhibitory effects on HCC cells. To test this hypothesis, we initially assessed the efficacy of the combined treatment in two LR HCC cell lines. Mono-treatment with lenvatinib alone was insufficient to inhibit the growth of the resistant cell lines, whereas the combination of both drugs significantly suppressed the growth of the two LR cell lines (Fig. 3A). Subsequently, we further investigated the synergistic effects of avapritinib and lenvatinib in four PT HCC cell lines. In C3A and SNU-398, which exhibit high expression of PDGFRA, the combination of avapritinib and lenvatinib demonstrated a robust synergistic inhibitory effect on tumor cell growth (Fig. 3B). However, when PDGFRA was knocked down in SNU398 and Huh7 cells, the synergistic inhibitory effect of avapritinib and lenvatinib was significantly attenuated (Supplementary Figure S4). The synergistic effects of the combination therapy involving avapritinib and lenvatinib were evaluated in both PDGFRA-high and PDGFRA-low HCC cell lines, including those exhibiting LR. The Bliss Independence Model was utilized to quantify the synergistic interactions between these two drugs in colony-formation assays. The results indicated that in HCC cells with high PDGFRA expression and LR, avapritinib and lenvatinib exhibited significant synergistic inhibitory effects (Supplementary Figure S5). In contrast, in SMMC-7721 and SNU-449 cells with relatively low PDGFRA expression, avapritinib and lenvatinib did not demonstrate significant synergistic inhibitory effects.

We then further validated the aforementioned results using mouse models. Upon subcutaneous tumors established from drug-resistant HCC cell lines, the combination of avapritinib and lenvatinib displayed a significant synergistic inhibition of subcutaneous tumor growth (Figs. 3C-E). Similarly, in subcutaneous tumors established from cell lines overexpressing PDGFRA, the combined treatment with avapritinib and lenvatinib also displayed an astonishing synergistic inhibitory effect (Figs. 3F-H). Importantly, the combined treatment with both drugs did not exhibit untoward toxicity to various organs including the heart, liver, spleen, lungs, and kidneys (Supplementary Figure S6). Thus, these results indicate that regardless of high PDGFRA expression or LR, avapritinib potently enhanced the sensitivity of these HCC cells to lenvatinib, thereby exhibiting a significant synergistic inhibitory effect.

### Verification of the synergistic antitumor effect of lenvatinib and avapritinib in an orthotopic HCC mouse model

**Fig. 4:**
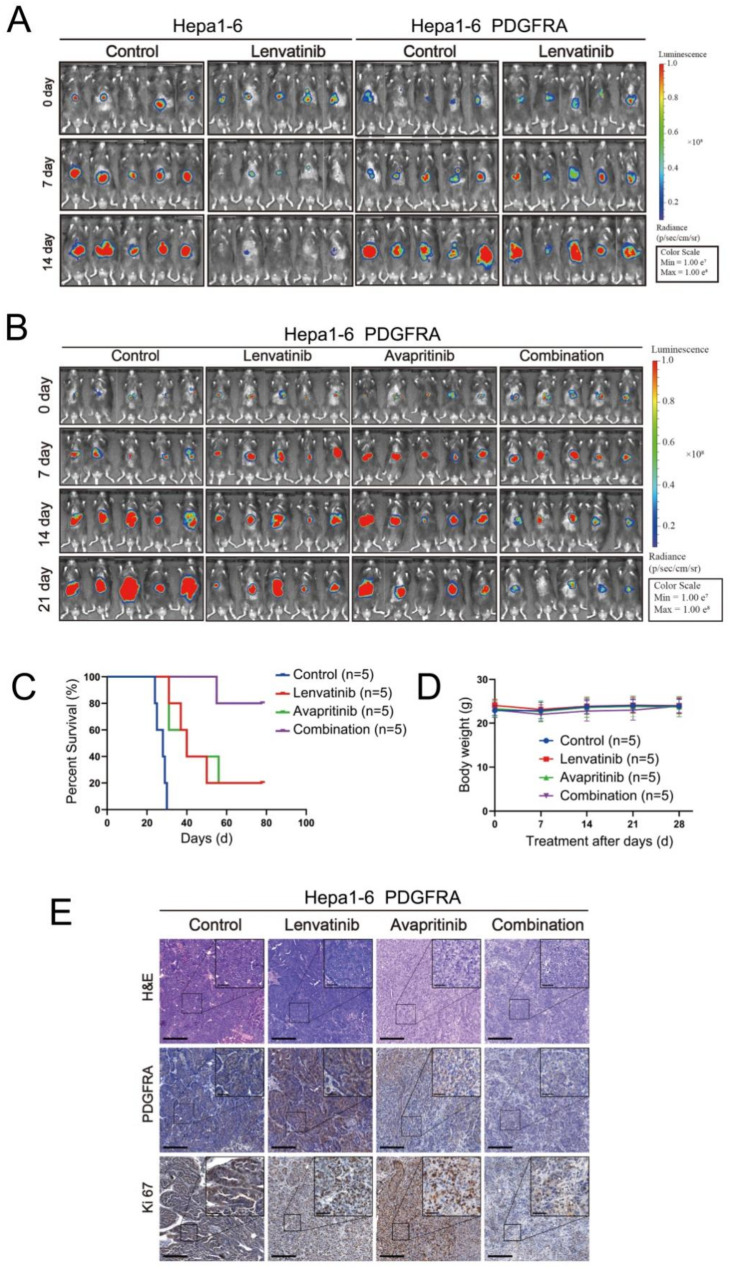
Mouse orthotopic HCC model reveals a synergistic activity of the avapritinib and Lenvatinib combination. (**A**) Mouse HCC orthotopic xenograft models were established with PDGFRA-overexpressing or control Hepa1-6-Luc cells and treated with lenvatinib or PBS as a control. Representative bioluminescent images of mice from the indicated groups are displayed. (**B**–**D**) Mice bearing PDGFRA-overexpressing Hepa1-6 orthotopic HCC models were treated with lenvatinib, avapritinib, or a combination of both. Representative bioluminescent images (**B**), survival curves (**C**), and body weight measurements (**D**) of mice from the indicated groups are presented. (**E**) Representative H&E staining and IHC images of PDGFRA and Ki67 in the orthotopic transplantation tumor model are shown

The aforementioned data have substantiated, both at the cellular level and using a murine subcutaneous xenograft model, the role of PDGFRA in conferring resistance to lenvatinib in HCC cells, and the capacity of avapritinib to enhance their sensitivity to this drug. To further corroborate our encouraging in vivo findings, we employed an orthotopic HCC mouse tumor model. Utilizing Hepa1-6 murine hepatoma cells, we established an orthotopic liver cancer model in mice and first administered lenvatinib as a monotherapeutic intervention. The findings revealed that in the control group of HCC tumors, lenvatinib treatment robustly curtailed tumor growth. In contrast, the therapeutic efficacy of lenvatinib in Hepa1-6 cells ectopically overexpressing PDGFRA, was markedly diminished, thereby reaffirming the pivotal role of elevated PDGFRA levels in mediating LR in HCC cells (Fig. 4A). Moreover, the synergistic therapeutic approach combining avapritinib with lenvatinib demonstrated a pronounced suppression of orthotopic HCC tumor growth and a concomitant enhancement in the overall survival rate of mice, underscoring the remarkable potential of this combination regimen in HCC treatment (Fig. 4B-E).

### PDO and PDX models confirm the synergistic effects of lenvatinib and avapritinib on HCC

**Fig. 5:**
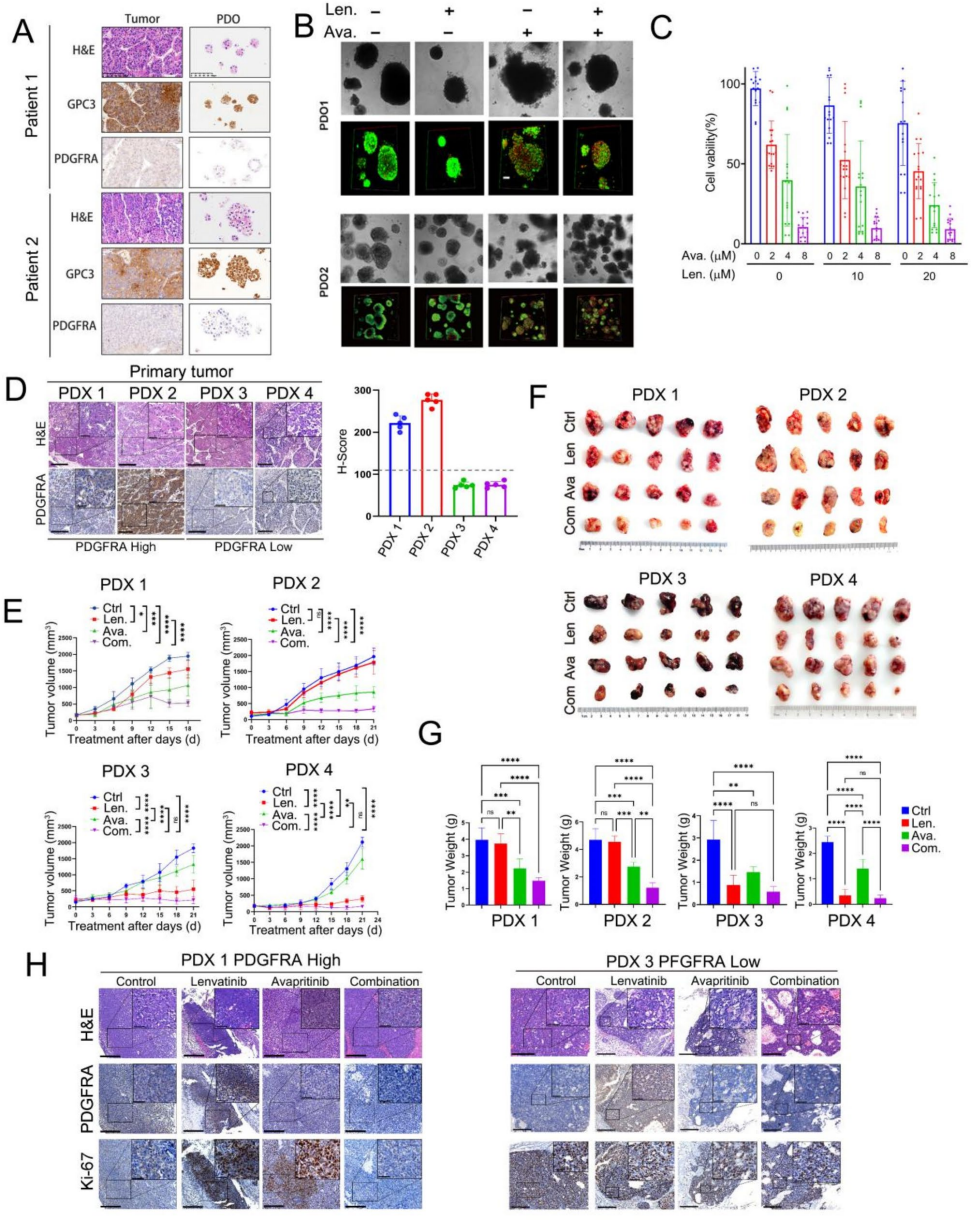
PDO and PDX models confirm the synergistic effects of Lenvatinib and Avapritinib on HCC. (**A**) Comparative histopathological features between HCC Patient-Derived Organoids (PDOs) and their corresponding original tumors, with representative H&E staining and IHC images for GPC3 and PDGFRA in both HCC PDOs and original tumors. (**B**) Live/dead cell imaging of PDOs following treatment with lenvatinib, avapritinib, or a combination of both, as indicated. (**C**) Quantitative analysis of cell viability in PDO models after treatment with lenvatinib and avapritinib. (**D**) Representative H&E staining and IHC images for PDGFRA and Ki67 in the Patient-Derived Xenograft (PDX) tumor model, with the right panel displaying the H-Score for PDGFRA in four PDX models. (**E**–**G**) PDX model mice were treated with lenvatinib, avapritinib, or a combination of both. Representative tumor growth curves (**E**) for xenograft tumors (**F**) and tumor weight (**G**) of PDX mice from the indicated treatment groups are presented. (**H**) Representative H&E staining and IHC images for PDGFRA and Ki67 in the PDX model are shown

We then further corroborated our findings using patient-derived organoid (PDO) and patient-derived xenograft (PDX) models. We first collected HCC specimens from newly diagnosed patients. The specimens were split into two portions that were processed for histological analysis or PDO establishment, allowing for a comprehensive characterization of the samples. After successful cultivation of PDO, histological analysis of paraffin-embedded sections was performed to explore whether the HCC PDOs preserved the histological features of the original tumors; the results showed that PDOs resembled those of the corresponding tumors and the expression of glypican 3 (GPC3), a well-established surface biomarker of HCC, exhibited the same expression pattern in the PDOs and original tumors (Fig. 5A-B). Notably, our findings revealed that the combination treatment with avapritinib and lenvatinib exerted a significant synergistic inhibitory effect on the growth of PDO samples (Fig. 5B, C). In contrast to PDO samples with high PDGFR expression, the combination of avapritinib and lenvatinib did not show any significant synergistic inhibitory effect in PDO samples displaying low PDGFR expression (Supplementary Figure S7). Moreover, upregulation of PDGFRA expression was detected in PDO samples treated with lenvatinib (Supplementary Figure S8), which is consistent with the findings in the cell and mouse models.

Subsequently, we explored whether PDGFRA mediates LR in patient-derived xenograft (PDX) mouse models. We initially selected tumor tissues from HCC patients to establish PDX models and assessed PDGFRA expression levels in the primary tumor tissues using immunohistochemical staining (Fig. 5D). Based on the histochemistry score (H-score), the HCC cases were classified into high PDGFRA expression (two cases) and low PDGFRA expression group (two cases). Thereafter, upon construction of PDX models, mice bearing tumors were treated with lenvatinib, avapritinib or a combination treatment. The results demonstrated that in PDX models with high PDGFRA expression, avapritinib treatment significantly inhibited tumor growth, whereas lenvatinib alone did not exhibit significant suppressive effects on tumor growth. Most importantly, the combination of both drugs further suppressed tumor growth (Fig. 5E-G). In contrast, in PDX models with low PDGFRA expression, the avapritinib and lenvatinib combination did not show any pronounced synergistic effect in inhibiting tumor growth (Fig. 5E-G). Monitoring mouse body weight indicated that treatment with either drug did not significantly affect the weight of mice in both high- and low PDGFRA-expressing PDX models (Supplementary Figure S9). In PDX tumor tissues, the expression of PDGFRA was reassessed using immunohistochemical analysis. Concurrently, immunostaining for the proliferation marker Ki-67 was employed to further substantiate the suppressive effects of lenvatinib and avapritinib on tumor cell proliferation (Fig. 5H and Supplementary Figure S10).

### High expression of PDGFRA is associated with poor prognosis in HCC patients

**Fig. 6:**
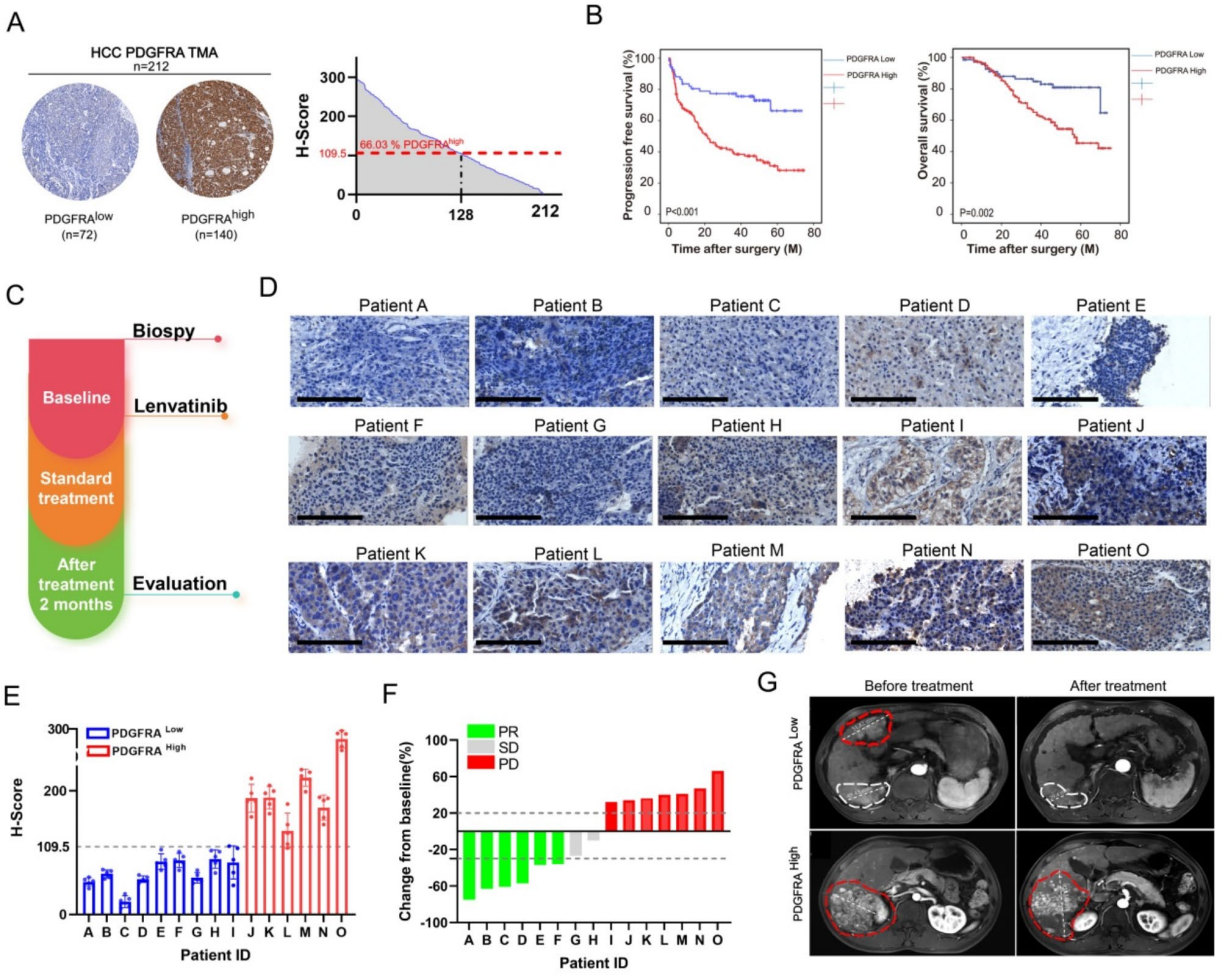
Correlation between PDGFRA Expression Levels and Response to Lenvatinib Treatment. (**A**) IHC detection of PDGFRA expression in a tissue microarray from 212 HCC cases, with the left panel showing a representative PDGFRA IHC image and the right panel illustrating the distribution of PDGFRA expression levels across various H-score ranges in 212 HCC patient specimens. (**B**) Kaplan-Meier survival analysis of the correlation between PDGFRA expression levels and overall survival and progression-free survival in a cohort of 212 HCC patients. (**C**) Schematic diagram of the treatment process for the retrospective analysis of 15 samples. (**D**-**E**) IHC staining images (**D**) and H-Score (**E**) of PDGFRA in liver cancer tissue samples from 15 patients. (**F**) Treatment response profiles for the 15 patients. (**G**) Representative magnetic resonance imaging (MRI) images of target lesions in Patients A (PDGFRA low) and Patients O (PDGFRA high) before and after treatment

Our findings corroborate the role of PDGFRA in HCC resistance to lenvatinib across cellular, organoid, as well as in vivo xenograft and orthotopic HCC tumor models in mice. Yet, the question remains whether PDGFRA parallels this association in the therapeutic response to lenvatinib in the clinical setting of HCC patients. Towards this end, we conducted an immunohistochemical analysis of PDGFRA expression in a cohort of 212 HCC tissue microarrays, stratifying the samples into high and low PDGFRA expression groups based on the H-Score system (Fig. 6A). Subsequent analysis of follow-up data indicated that elevated PDGFRA expression was associated with diminished overall and progression-free survival rates in HCC patients (Fig. 6B). Both univariate and multivariate Cox regression analyses identified PDGFRA H-score as an independent prognostic factor for relapse-free and overall survival in liver cancer patients (Supplementary Table S1).

We further examined liver tissue biopsies from 15 HCC patients who had undergone lenvatinib combination therapy (Fig. 6C and Supplementary Table S2). All 15 HCC patients were administered Transarterial Chemoembolization (TACE) in conjunction with lenvatinib. A subset of these patients also received a combination therapy consisting of TACE, lenvatinib, and an anti-PD-1 monoclonal antibody; the specific details regarding the treatment regimens are delineated in Supplementary Table S2. Based on the outcomes of immunohistochemical staining, the cohort of 15 patients was stratified into two groups: one with high PDGFRA expression and another with low PDGFRA levels (Fig. 6D-E). Correlation with the patients’ therapeutic responses to lenvatinib revealed that all 7 patients with high PDGFRA expression experienced disease progression (PD), whereas among those with low PDGFRA expression, 5 demonstrated partial response (PR), and 2 exhibited stable disease (SD) (Fig. 6F). Radiological data provided illustrative examples of the typical treatment response profiles for both patient groups (Fig. 6G). Collectively, these findings suggest that elevated PDGFRA expression in liver cancer tissues is associated with reduced sensitivity to lenvatinib therapy, underscoring the potential of PDGFRA expression as a predictive biomarker for the therapeutic response to lenvatinib in HCC patients.

### The PTEN/AKT/GSK-3β/β-catenin signaling axis is implicated in the drug resistance mechanism

**Fig. 7:**
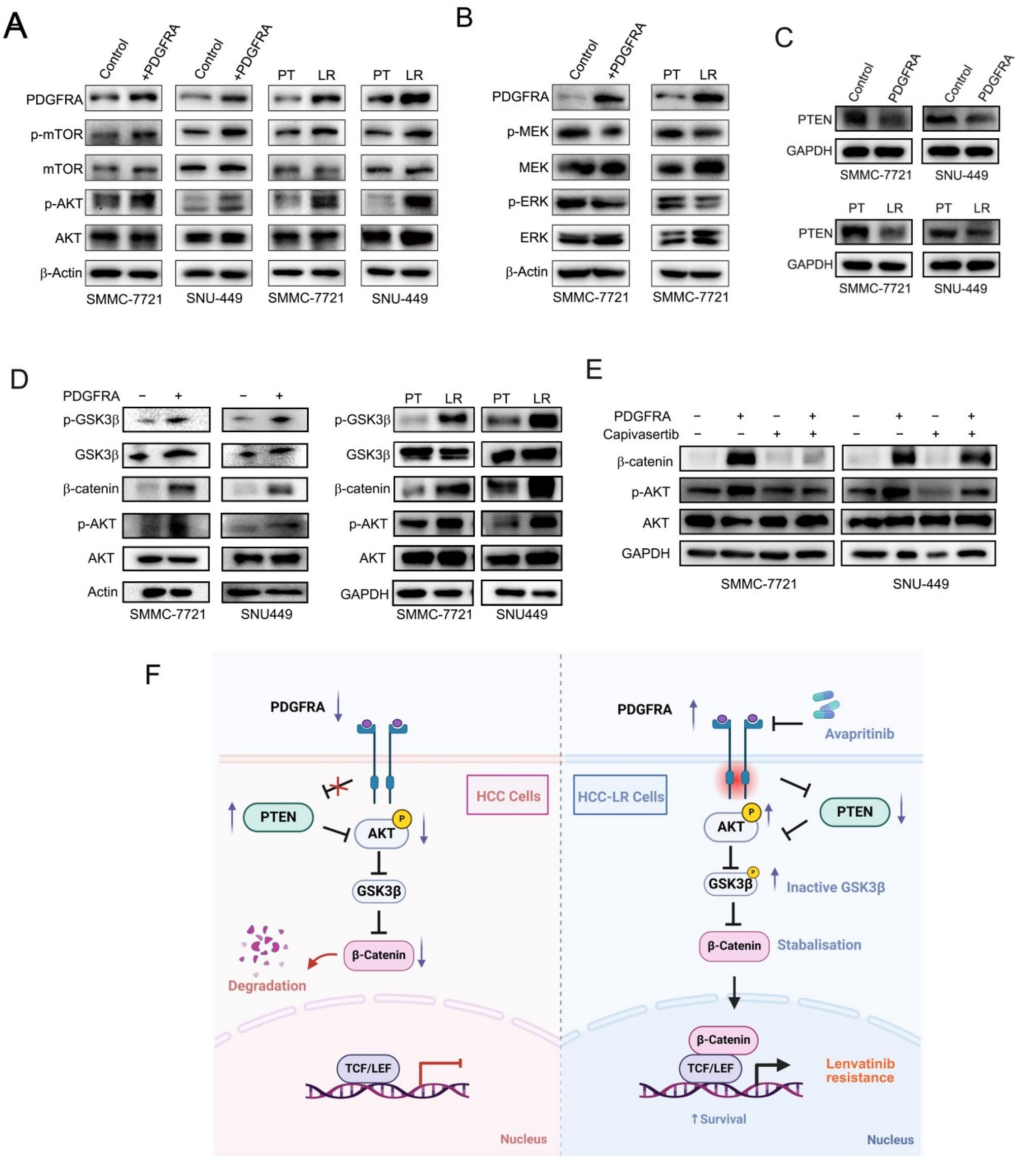
PDGFRA Mediates Lenvatinib Resistance in HCC Cells through the PTEN/AKT/GSK-3β/β-catenin Signaling Axis. (**A**) Protein levels of AKT pathway genes in PDGFRA-overexpressing or lenvatinib-resistant HCC cells. (**B**) Protein levels of MEK and ERK in PDGFRA-overexpressing or lenvatinib-resistant HCC cells. (**C**) Western blot analysis of PTEN protein levels in PDGFRA-overexpressing and lenvatinib-resistant HCC cells. (**D**) Impact of PDGFRA overexpression and lenvatinib resistance on the GSK-3β/β-catenin signaling pathway. (**E**) Effects of PDGFRA overexpression on AKT, phospho-AKT (p-AKT), and β-catenin under treatment with the AKT inhibitor capivasertib. (**F**) Schematic representation of PDGFRA-mediated lenvatinib resistance in HCC through the PTEN/AKT/GSK-3β/β-catenin signaling axis

To further elucidate the molecular mechanisms by which PDGFRA mediates lenvatinib resistance in HCC cells, we conducted additional experiments to assess the expression of downstream targets of the PDGF/PDGFRA signaling pathway, including mTOR, AKT, MEK, and ERK. Our results indicated that in cell lines with overexpression of PDGFRA and LR, phosphorylation of AKT was significantly upregulated (Fig. 7A, B). Numerous studies have suggested that PTEN functions as a tumor suppressor by negatively regulating the AKT/PKB signaling pathway [17]. We further examined the expression of PTEN, and found that PDGFRA significantly downregulated the expression of PTEN, and its expression was also significantly suppressed in resistant cell lines (Fig. 7C). The above results suggest that lenvatinib upregulates PDGFRA, thereby activating the PTEN-AKT signaling pathway. The AKT/GSK3β/β-catenin pathway has been implicated in tumor proliferation, invasion, metastasis, stemness, and drug resistance, including LR [18]. Consequently, we further examined the expression of downstream targets of this signaling pathway, namely GSK3β and β-catenin. Our results are consistent with our predictions, showing significant upregulation of p-GSK3β and β-catenin expression in both LR cell lines and cells with PDGFRA overexpression (Fig. 7D). To further substantiate the involvement of the AKT signaling pathway in PDGFRA-mediated activation of the GSK3β/β-catenin pathway, we treated cells with capivasertib, an FDA approved selective inhibitor of AKT [19]. Our results demonstrated that in the presence of AKT inhibition, the activating effect of PDGFRA on β-catenin was significantly diminished, suggesting that PDGFRA regulates the GSK3β/β-catenin pathway through AKT signaling (Fig. 7E). Therefore, through the aforementioned molecular mechanistic studies, we conclude that lenvatinib treatment leads to upregulation of PDGFRA, which in turn activates the PTEN/AKT/GSK3β/β-catenin signaling axis, thereby promoting resistance to lenvatinib (Fig. 7F).

## Discussion

Lenvatinib is a multiple kinase inhibitor serving as a first-line anticancer agent for advanced HCC that cannot be surgically removed in patients who have not been treated with prior chemotherapy; it has demonstrated notable antitumor efficacy [20]. However, the emergence of lenvatinib resistance limits its therapeutic efficacy, adversely affecting patient survival [21]. A plethora of recent studies has unveiled various determinants contributing to lenvatinib resistance including activation of the epidermal growth factor receptor (EGFR), overexpression of fibroblast growth factor receptor 1 (FGFR1), the emergence of cancer stem cells, a hypoxic microenvironment, an immunosuppressive microenvironment, extracellular matrix remodeling, and metabolic reprogramming [22]. For example, the feedback activation of EGFR has been demonstrated to attenuate lenvatinib efficacy, and the combinatorial application of EGFR inhibitors with lenvatinib surmounts tumor drug resistance [23]. This latter study revealed that using a kinome-based CRISPR/Cas9 genetic screen, it was discovered that inhibition of EGFR is synthetic lethal with Lenvatinib treatment of liver cancer. Our current investigation has unveiled that PDGFRA mediates resistance to lenvatinib. Most significantly, research conducted across cellular, animal, PDO and PDX models has consistently revealed that the concomitant use of the PDGFRA inhibitor avapritinib potentiates the activity of lenvatinib. Furthermore, we have corroborated the correlation between PDGFRA levels and treatment response in lenvatinib-treated liver cancer patients through patient biopsy samples, proposing that the targeting of PDGFRA could represent an efficacious strategy to surmount lenvatinib resistance.

Lenvatinib has been shown to inhibit HCC angiogenesis, a critical process in cancer progression [24, 25]. Despite the initial efficacy of lenvatinib, the frequent emergence of lenvatinib resistance is a common clinical challenge, diminishing its therapeutic potential as well as patient survival. Deciphering mechanisms of resistance to lenvatinib and other chemotherapeutics as well as identifying biomarkers associated with drug resistance have emerged as central areas of cancer research in recent years [2, 26–29]. As a multiple kinase inhibitor, resistance to lenvatinib has been linked to the compensatory activation of downstream targets in drug resistant cells; these include VEGFR [30], RET [31], FGFR [32], and KIT [33]. Inhibitors of these targets represent promising strategies to overcome lenvatinib resistance. Our current research has demonstrated for the first time that PDGFRA is activated in lenvatinib-resistant cells, and targeting PDGFRA is an efficacious modality to surmount lenvatinib resistance. Furthermore, several other resistance targets and pathways have been previously implicated in lenvatinib resistance. These include the p-MYH9/USP22/HIF-1α signaling pathway [34], ITGB8/HSP90/AKT axis [35], as well as FZD10 [6]. Our current study also indicates that upregulation of PDGFRA activates the AKT signaling pathway. This finding reinforces the potential of AKT inhibitors as a strategy to overcome lenvatinib resistance.

Avapritinib, a potent and highly selective inhibitor of mutated KIT and PDGFRA kinases, is an approved agent for the treatment of GIST, given that up to 85% of patients present mutations in either the PDGFRA or KIT genes [36]. Furthermore, systemic mastocytosis (SM), a rare condition, is characterized by a KIT D816V mutation in approximately 95% of cases. The FDA has granted approval for avapritinib in the treatment of GIST and advanced SM. Beyond these indications, avapritinib has not received FDA approval for any additional indications. Structural studies of the PDGFRA protein have demonstrated that avapritinib is capable of binding to the wild-type PDGFRA [37]. This finding is in accord with our finding in our current research where upregulation of PDGFRA expression was associated with lenvatinib resistance, and the concurrent use of avapritinib enhanced the sensitivity to lenvatinib. Consequently, for patients with HCC who exhibit primary (intrinsic) or secondary (acquired) resistance to lenvatinib, the combination therapy with both Lenvatinib and avapritinib may prove a potent treatment strategy. Our study also offers evidence supporting the broadening the therapeutic applications of avapritinib.

The present study acknowledges its inherent limitations. Our research identified a marked increase in PDGFRA expression in cells exhibiting lenvatinib resistance. Further exploration into PDGFRA mutations and copy number variations did not reveal any mutations or gene amplification. As a result, the specific mechanism responsible for the upregulation of PDGFRA in lenvatinib-resistant cells remains unknown and necessitates additional research. While resistance to specific kinase inhibitors is frequently linked to mutations in the target protein [38], this is not the exclusive mechanism of drug resistance in cancer cells. Compensatory alterations in the signaling pathways of drug treated cancer cells circumvent drug-mediated inhibition, and such compensatory mechanisms significantly contribute to drug resistance, especially to multi-kinase inhibitors [39]. For example, prolonged sorafenib treatment and its anti-angiogenic effects result in reduced microvascular density, which promotes tumor hypoxia and hypoxia-inducible factor (HIF)-mediated cellular responses. These responses lead to the compensatory activation of a range of downstream targets of HIF, contributing to sorafenib resistance in HCC [40]. Moreover, several studies have reported compensatory activation of EGFR in lenvatinib-resistant HCC cells [23, 41, 42]. Consequently, we hypothesize that PDGFRA may also be activated through compensatory mechanisms in lenvatinib-resistant HCC cells.

## Conclusions

In summary, our study uncovers that the upregulation of PDGFRA confers resistance to lenvatinib therapy in HCC cells via the PTEN/AKT/GSK-3β/β-catenin signaling cascade. Notably, the PDGFRA-targeting agent avapritinib has been shown to enhance the sensitivity of HCC cells to lenvatinib across cellular assays, patient-derived organoid cultures, and diverse animal models. Additionally, we have corroborated a significant correlation between PDGFRA expression and patient response to lenvatinib treatment in clinical patient samples.

## Electronic supplementary material

Below is the link to the electronic supplementary material.

Supplementary Material 1

## Data Availability

The data that support the findings of this study are available from the corresponding author upon reasonable request.
