# Correction: Targeted Inhibition of PDGFRA with avapritinib, markedly enhances lenvatinib efficacy in hepatocellular carcinoma in vitro and in vivo: clinical implications

**DOI:** 10.1186/s13046-025-03423-6

**Published:** 2025-05-26

**Authors:** Bixing Zhao, Yang Zhou, Niangmei Cheng, Xiaoyuan Zheng, Geng Chen, Xin Qi, Xiangzhi Zhang, Fei Wang, Qiuyu Zhuang, Yehuda G. Assaraf, Xiaolong Liu, Yingchao Wang, Yongyi Zeng

**Affiliations:** 1 The United Innovation of Mengchao Hepatobiliary Technology Key Laboratory of Fujian Province, Mengchao Hepatobiliary Hospital of Fujian Medical University, Fuzhou, 350025 P. R. China; 2 Mengchao Med-X Center, Fuzhou University, Fuzhou, 350116 P. R. China; 3 The Fred Wyszkowski Cancer Research Laboratory, Faculty of Biology, Technion-Israel Institute of Technology, Haifa, 3200003 Israel

**Correction: J Exp Clin Cancer Res 44**,** 139 (2025)**

10.1186/s13046-025-03386-8

Following the publication of the original article [1], the authors identified an identical images mistakenly used in Fig. 3A and B. The figure figure is presented below:

**Incorrect Fig. 3**

**Fig. 3:**
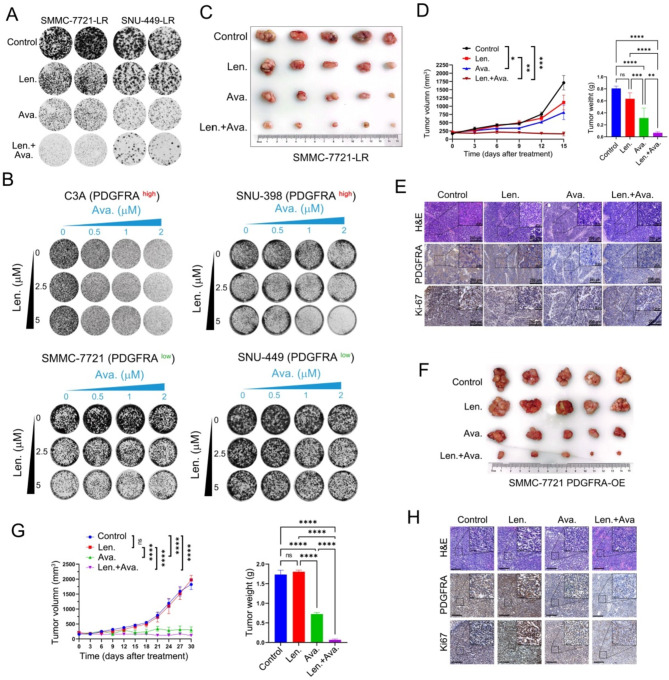
PDGFRA inhibitor avapritinib sensitizes HCC cells to lenvatinib treatment. (**A**) The effect of lenvatinib and avapritinib was tested in the colonyformation assay in SMMC-7721-LR and SNU-449-LR cells. (**B**) Synergistic response to the combination of lenvatinib and avapritinib in PDGFRA high C3A and SNU-398 cells and in PDGFRA low SMMC-7721 and SNU-449 cells. (**C**) Representative tumor images of each group of SMMC-7721-LR xenografts at the end of lenvatinib, avapritinib or combination treatment. (**D**) Tumor growth curves and tumor weight of each group are shown. (**E**) Representative H&E staining, IHC images of PDGFRA and Ki67 in subcutaneous implantation mouse model. (**F**) Representative tumor images of each group of PDGFRA overexpressing SMMC-7721 xenografts at the end of lenvatinib, avapritinib or combination treatment. (**G**) Tumor growth curve and tumor weight of each group (H) Representative H&E staining, IHC images of PDGFRA and Ki67 in subcutaneous implantation mouse model

**Correct Fig. 3**

**Fig. 3:**
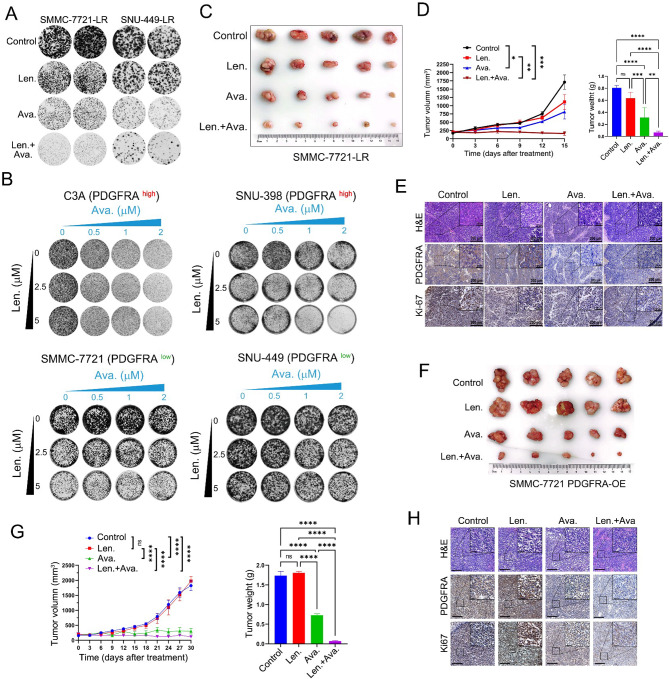
PDGFRA inhibitor avapritinib sensitizes HCC cells to lenvatinib treatment. (**A**) The effect of lenvatinib and avapritinib was tested in the colonyformation assay in SMMC-7721-LR and SNU-449-LR cells. (**B**) Synergistic response to the combination of lenvatinib and avapritinib in PDGFRA high C3A and SNU-398 cells and in PDGFRA low SMMC-7721 and SNU-449 cells. (**C**) Representative tumor images of each group of SMMC-7721-LR xenografts at the end of lenvatinib, avapritinib or combination treatment. (**D**) Tumor growth curves and tumor weight of each group are shown. (**E**) Representative H&E staining, IHC images of PDGFRA and Ki67 in subcutaneous implantation mouse model. (**F**) Representative tumor images of each group of PDGFRA overexpressing SMMC-7721 xenografts at the end of lenvatinib, avapritinib or combination treatment. (**G**) Tumor growth curve and tumor weight of each group (H) Representative H&E staining, IHC images of PDGFRA and Ki67 in subcutaneous implantation mouse model

The correction does not compromise the validity of the conclusions and the overall content of the article. The original article [1] has been updated.

## References

[1] Zhao B, Zhou Y, Cheng N, et al. Targeted Inhibition of PDGFRA with avapritinib, markedly enhances lenvatinib efficacy in hepatocellular carcinoma in vitro and in vivo: clinical implications. J Exp Clin Cancer Res. 2025;44:139. 10.1186/s13046-025-03386-8. 40336047 10.1186/s13046-025-03386-8 PMC12057143

