# Supplementary material for: Targeted inhibition of PDGFRA with avapritinib, markedly enhances lenvatinib efficacy in hepatocellular carcinoma in vitro and in vivo: clinical implications
Source: J Exp Clin Cancer Res. 2025 May 7;44:139. doi: 10.1186/s13046-025-03386-8 (PMC12057143; doi:10.1186/s13046-025-03386-8)
Supplement: Supplementary file 1 — Supplementary Material 1 [file 13046_2025_3386_MOESM1_ESM.docx]

**Supplementary Materials**

**Supplementary Figures**


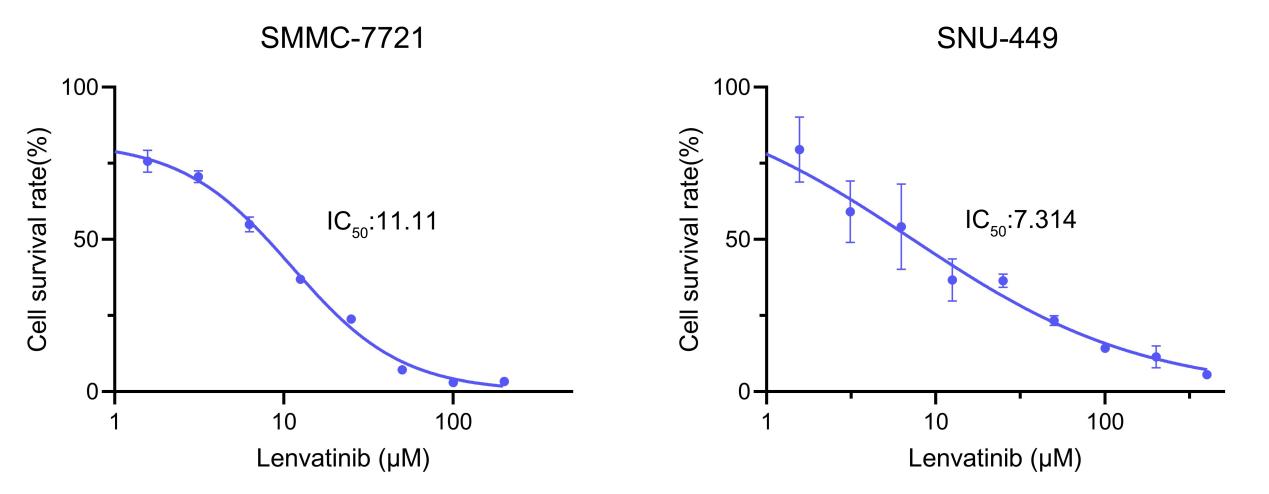


**Supplementary Figure S1.** Half maximal inhibitory lenvatinib concentration curves of SMMC-7721 and SNU-449 cells after treatment with lenvatinib for 3 days


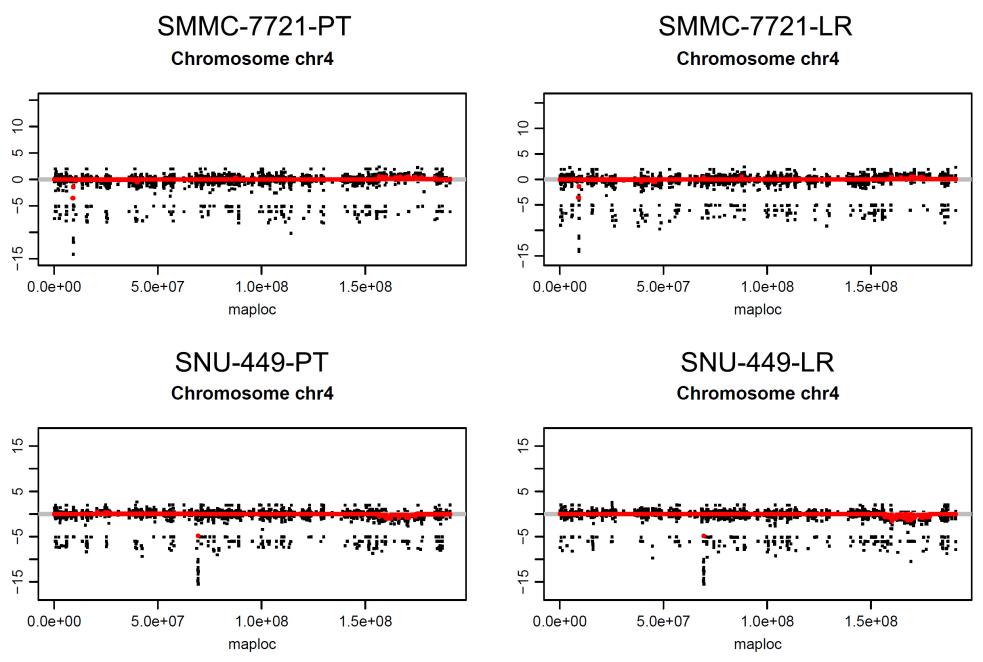


**Supplementary Figure S2.** Analysis of Copy Number Variations (CNVs) in Four Cell Lines. A comparative assessment of copy number variations was conducted among the four cell lines: SMMC-7721-PT, SMMC-7721-LR, SNU-449-PT, and SNU-449-LR. Notably, the region harboring the PDGFRA gene on chromosome 4 showed no significant alterations in copy number across these cell lines.


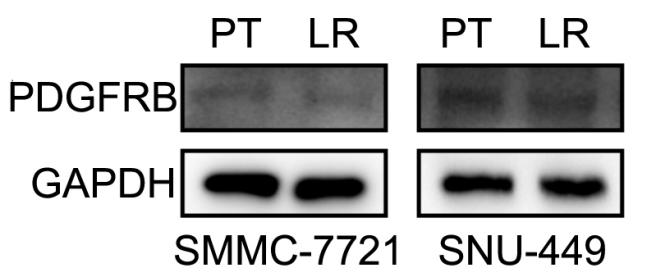


**Supplementary Figure S3.** Expression of PDGFRβ in Four Cell Lines Assessed by Western Blot. The protein expression levels of PDGFRβ were examined in the SMMC-7721-PT, SMMC-7721-LR, SNU-449-PT, and SNU-449-LR cell lines using Western blot analysis.

**
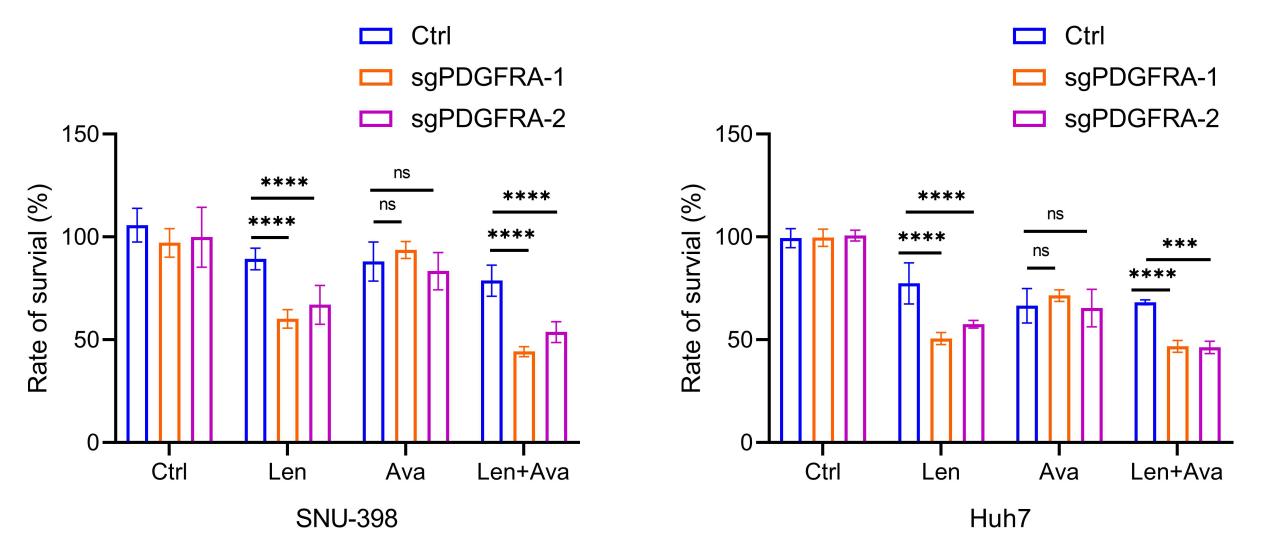
**

**Supplementary Figure S4: Synergistic response to the combination of lenvatinib and avapritinib in PDGFRA knockdown SNU-398 and Huh7 cells.**

**
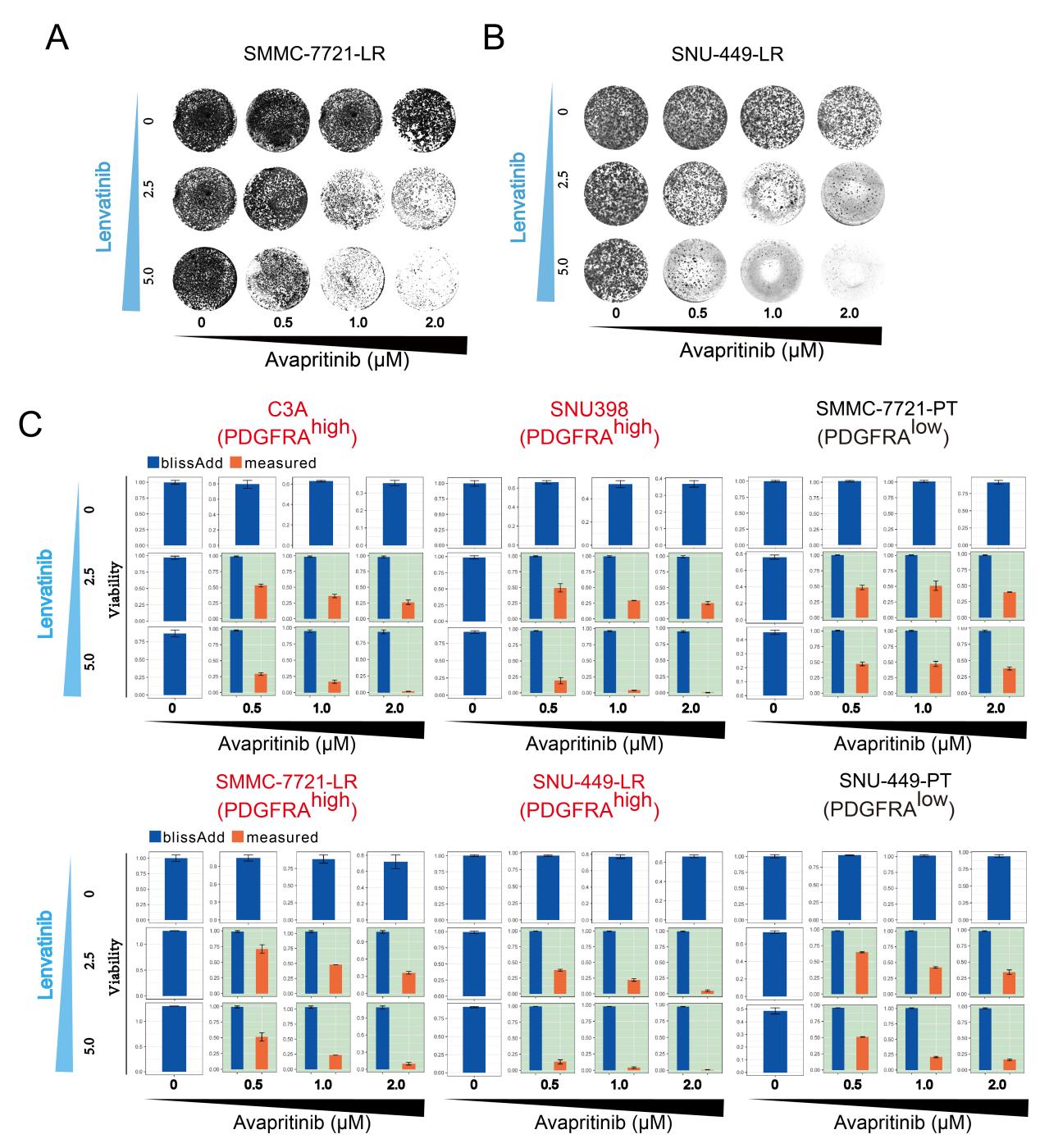
**

**Supplementary Figure S5.** Bliss independence model evaluating the synergistic effect between lenvatinib with avapritinib HCC cells. (A-B) Synergistic response to the combination of lenvatinib and avapritinib in SMMC-7721-LR (A) and SNU-449-LR cells (B) tested in the colony-formation assay. (C) The Bliss independence model was utilized to analyze the colony-formation experiment data presented in Figure 3B and Supplementary Figure S4A-B, assessing the synergistic effects of lenvatinib and avapritinib. In the untreated condition (absence of both lenvatinib and avapritinib), cell viability was normalized to 0, indicating no inhibition. Additive scores (blissAdd) were calculated by multiplying the normalized effects of the two individual drugs. To evaluate the synergistic effect, we computed the reduction in viability by subtracting the mean blissAdd from the mean measured combination scores. A green background highlights areas of synergy, defined as P ≤ 0.05 and a reduction in viability exceeding 10%.


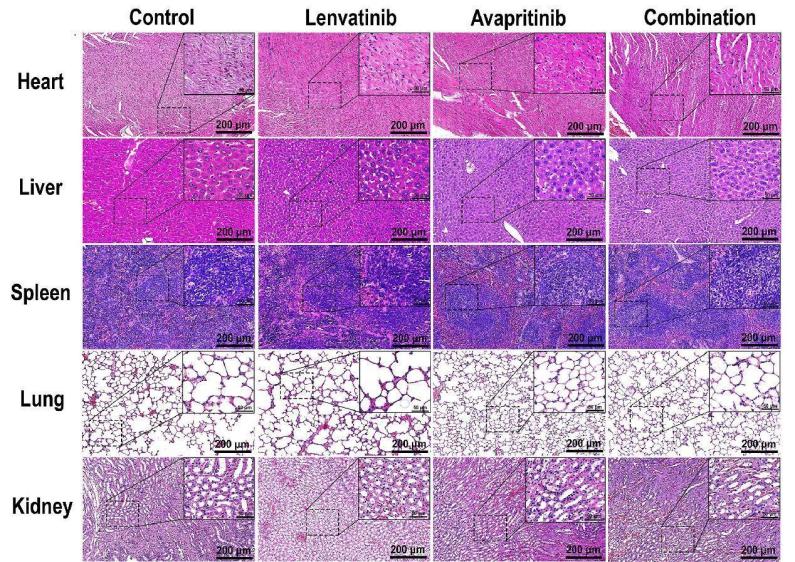


**Supplementary Figure S6.** H&E staining for vital organs in mice treated with lenvatinib, avapritinib, or a combination of both. Scale bar: 200 μm

**
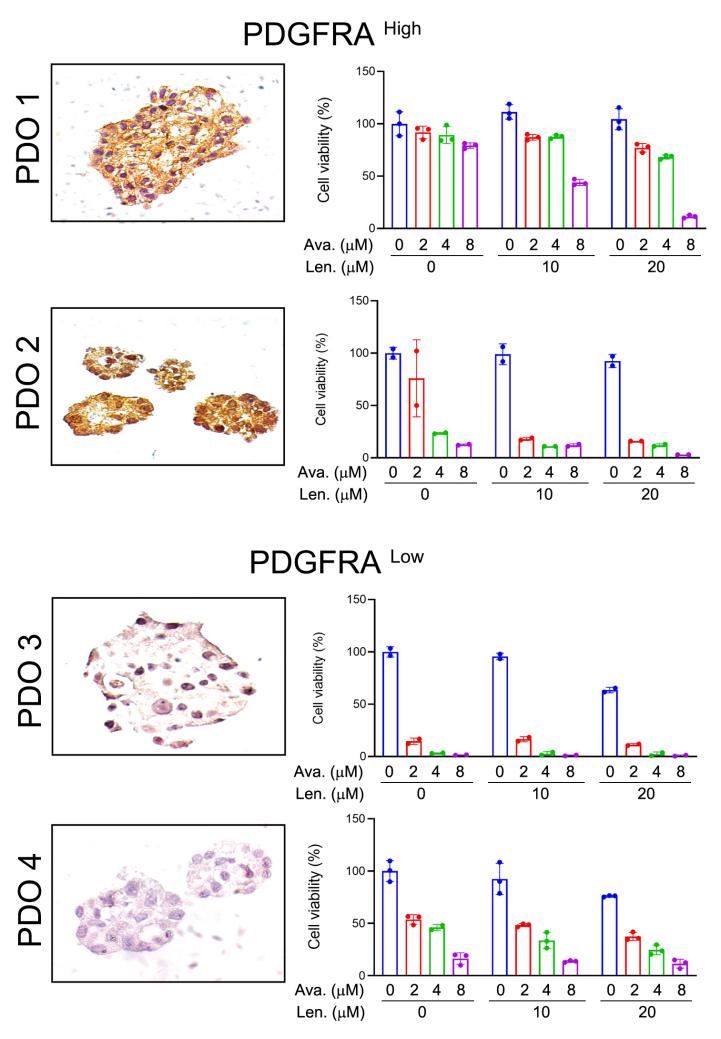
**

**Supplementary Figure S7. Synergistic effects of Lenvatinib and Avapritinib on PDGFRA high and PDGFRA low PDO**

**
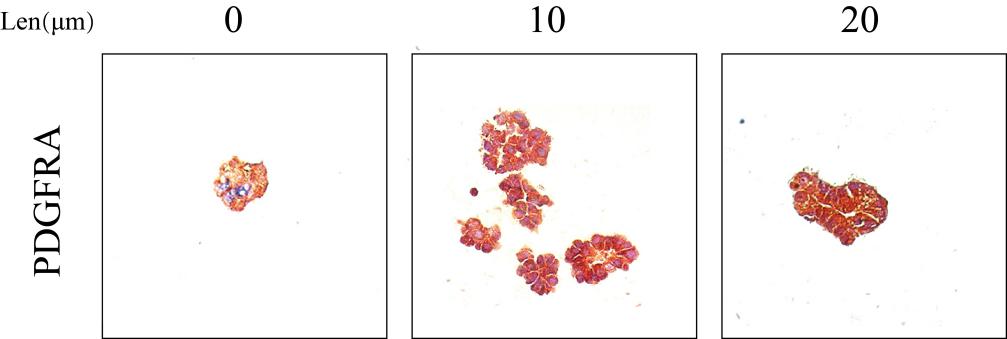
**

**Supplementary Figure S8. The expression of PDGFRA in PDO samples after treatment with Lenvatinib.**


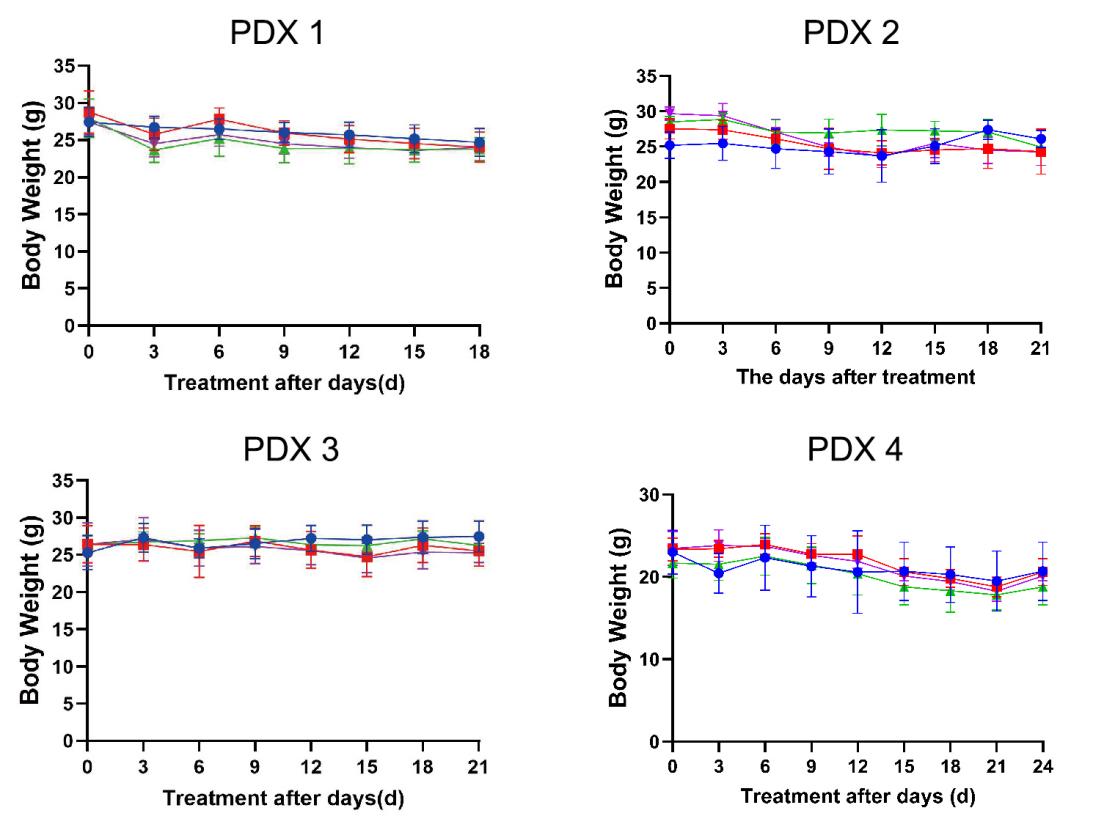


**Supplementary Figure S9.**  Body weight measurements of mice from 4 PDX model mice.


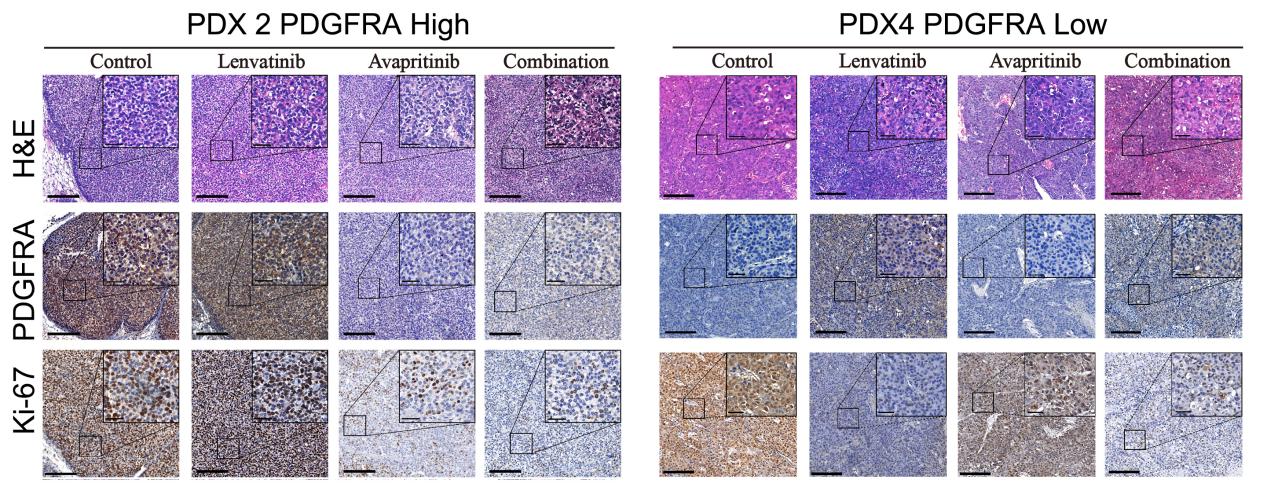


**Supplementary Figure S10.** Representative H&E staining and IHC images for PDGFRA and Ki67 in the PDX model are shown.

**Supplementary Table**

**Supplementary Table S1.** Univariate and multivariate Cox regression analysis revealed that PDGFRA H-score could be considered as an independent risk factor associated with Progression-Free Survival (PFS) and overall survival (OS) among HCC patients.

| **OS Characteristics** | **Univariate analysis** | | **Multivariate analysis** | |
| --- | --- | --- | --- | --- |
|  | HR（95% CI） | **P Value** | HR（95% CI） | **P Value** |
| H-score (PDGFRA High vs Low) | 2.447  (1.366-4.382) | 0.003 | 3.113 (1.632-5.939) | 0.001 |
| MVI (Present vs Absent) | 3.089  (1.526-6.253) | 0.002 | 2.623  (1.192-5.775) | 0.017 |

| **PFS Characteristics** | **Univariate analysis** | | **Multivariate analysis** | |
| --- | --- | --- | --- | --- |
|  | **HR（95% CI）** | **P Value** | **HR（95% CI）** | **P Value** |
| H-score (High expression vs Low expression) | 2.749 (1.684-4.489) | ＜0.0001 | 3.999 (2.360-6.777) | ＜0.001 |
| MVI （Present vs Absent） | 2.474 (1.427-4.288) | 0.001 | 2.489 (1.384-4.477) | 0.002 |
| Vascular Invasion （Present vs Absent） | 2.477 (1.484-4.132) | 0.001 | 2.729 (1.521-4.897) | 0.001 |
| Tumor number （Multiple vs Solitary） | 1.850 (1.212-2.822) | 0.004 | 1.595 (1.025-2.480) | 0.038 |
| Diameter of Tumor | 1.846 (1.234-2.761) | 0.003 | 1.542 (1.003-2.369) | 0.048 |
| （≥5 vs <5, cm） |  |  |  |  |

**Supplementary Table S2.** Information of 15 Patients Receiving Lenvatinib Therapy

| **Patient**  **ID** | **Gender** | **Age** | **Tumor: H-score** | **Treatment method** | **Response evaluation** | **Change from baseline(%)** |
| --- | --- | --- | --- | --- | --- | --- |
| A | male | 62 | 54.05 | TACE+Lenvatinib | PR | -75% |
| B | male | 70 | 67.95 | TACE+Lenvatinib | PR | -63% |
| C | male | 61 | 12.71 | TACE+Lenvatinib+Camrelizumab | PR | -61% |
| D | male | 60 | 63.98 | TACE+Lenvatinib+Pembrolizumab | PR | -57% |
| E | male | 60 | 83.33 | TACE+Lenvatinib | SD | -37% |
| F | male | 66 | 101.52 | TACE+Lenvatinib+Tislelizumab | PR | -36% |
| G | male | 58 | 71.58 | TACE+Lenvatinib+Camrelizumab | PR | -32% |
| H | male | 72 | 89.75 | TACE+Lenvatinib | SD | -10% |
| I | male | 47 | 88.54 | TACE+Lenvatinib+Camrelizumab | PD | 32% |
| J | female | 63 | 182.99 | TACE+Lenvatinib | PD | 34% |
| K | male | 60 | 196.16 | TACE+Lenvatinib | PD | 36% |
| L | male | 73 | 113.78 | TACE+Lenvatinib+Toripalimab | PD | 40% |
| M | male | 43 | 224.23 | TACE+Lenvatinib | PD | 41% |
| N | male | 46 | 184.7 | TACE+Lenvatinib+Camrelizumab | PD | 47% |
| O | male | 50 | 292.92 | TACE+Lenvatinib | PD | 66% |
